# *Francisella tularensis* subsp. *holarctica* Releases Differentially Loaded Outer Membrane Vesicles Under Various Stress Conditions

**DOI:** 10.3389/fmicb.2019.02304

**Published:** 2019-10-10

**Authors:** Jana Klimentova, Ivona Pavkova, Lenka Horcickova, Jan Bavlovic, Olga Kofronova, Oldrich Benada, Jiri Stulik

**Affiliations:** ^1^Department of Molecular Pathology and Biology, Faculty of Military Health Sciences, University of Defense, Hradec Kralove, Czechia; ^2^Institute of Microbiology of the Czech Academy of Sciences, Prague, Czechia; ^3^Faculty of Science, Jan Evangelista Purkyně University, Ústí nad Labem, Czechia

**Keywords:** *Francisella tularensis*, FSC200, outer membrane vesicles, stress response, virulence factor, host–pathogen interaction

## Abstract

*Francisella tularensis* is a Gram-negative, facultative intracellular bacterium, causing a severe disease called tularemia. It secretes unusually shaped nanotubular outer membrane vesicles (OMV) loaded with a number of virulence factors and immunoreactive proteins. In the present study, the vesicles were purified from a clinical isolate of subsp. *holarctica* strain FSC200. We here provide a comprehensive proteomic characterization of OMV using a novel approach in which a comparison of OMV and membrane fraction is performed in order to find proteins selectively enriched in OMV vs. membrane. Only these proteins were further considered to be really involved in the OMV function and/or their exceptional structure. OMV were also isolated from bacteria cultured under various cultivation conditions simulating the diverse environments of *F. tularensis* life cycle. These included conditions mimicking the milieu inside the mammalian host during inflammation: oxidative stress, low pH, and high temperature (42°C); and in contrast, low temperature (25°C). We observed several-fold increase in vesiculation rate and significant protein cargo changes for high temperature and low pH. Further proteomic characterization of stress-derived OMV gave us an insight how the bacterium responds to the hostile environment of a mammalian host through the release of differentially loaded OMV. Among the proteins preferentially and selectively packed into OMV during stressful cultivations, the previously described virulence factors connected to the unique intracellular trafficking of *Francisella* were detected. Considerable changes were also observed in a number of proteins involved in the biosynthesis and metabolism of the bacterial envelope components like O-antigen, lipid A, phospholipids, and fatty acids. Data are available via ProteomeXchange with identifier PXD013074.

## Introduction

*Francisella tularensis* is a Gram-negative, facultative intracellular bacterium, causing a severe disease known as tularemia. Being one of the most infectious pathogenic bacterium (as few as 10 bacteria can initiate the disease), *F. tularensis* has been classified as a potential biological warfare agent by the Working Group on Civilian Biodefense (Dennis et al., 2001). *F. tularensis* is transmitted to humans *via* inhalation, ingestion of contaminated food or water, bites by arthropods, or through direct contact with infected animals. Three different *F. tularensis* subspecies are distinguished which differ in their geographical distribution, virulence, and severity of the disease. Subsp. *tularensis* (type A) is found almost exclusively in North America and is characterized by the highest virulence, severity of the disease, and mortality. Subsp. *holarctica* (type B) is spread along the north hemisphere and exhibits moderate virulence. The last subsp. *mediasiatica* exhibits comparable virulence and is found in central Asia (Oyston, 2008).

*Francisella* has a broad range of environmental reservoirs with quite a high number of potential cold-blooded hosts (insects, arthropods, and fresh-water protozoans) as well as homeothermic hosts (rodents, lagomorphs, and other mammals including human), which indicates the remarkable adaptability of the bacterium (Hazlett and Cirillo, 2009; Zellner and Huntley, 2019). Transition of the bacterium from an environmental compartment or a vector into the mammalian host requires adaptation to new hostile conditions characterized by higher temperature, presence of reactive oxygen species, low pH, lack of iron, and limitations in nutrients. *Francisella* actively invades a wide range of host cells, macrophages, and dendritic cells being the primary phagocytic targets (Celli and Zahrt, 2013). After the engulfment, the bacterium alters maturation of the vacuole by a yet unexplained mechanism, decomposes the phagosomal membrane, and escapes into the cytosol where it multiplies (Clemens and Horwitz, 2007; Jones et al., 2012; Celli and Zahrt, 2013). Proteomic response of *Francisella* to various stress conditions like high temperature, stationary phase, iron restriction, or oxidative stress were comprehensively studied previously and revealed activation of number of virulence factors (Lenco et al., 2005, 2007, 2009).

Release of OMV is an alternative way of protein secretion that is common to all Gram-negative bacteria and it is independent of the secretion systems I–VI. OMV are 20–300 nm, usually spherical, double-layered membranous particles that are released from the bacterial OM. They are formed when a portion of OM separates and encapsulates part of the periplasmic space with its contents. As such they contain OM and periplasmic proteins, phospholipids, LPS, and peptidoglycan. Nevertheless, they can also harbor nucleic acids and cytosolic or inner membrane proteins (Ellis and Kuehn, 2010; Haurat et al., 2015). OMV have numerous functions in the bacterial lifestyle, and they can act both in their defense and offense (Kulp and Kuehn, 2010; Olsen and Amano, 2015). OMV serve as a delivery vehicle for insoluble or degradable material, for toxins and other virulence factors, DNA, or small molecules for bacterial communication. The release of misfolded or aggregated proteins in OMV has also been presented as an envelope-stress response mechanism (McBroom and Kuehn, 2007). In some bacteria OMV serve as a decoy for bacteriophages or antimicrobial peptides (Manning and Kuehn, 2011) and they present an important component of biofilms where they contribute to communication between bacteria and surfaces attachment (Baumgarten et al., 2012; Cooke et al., 2019). OMV also play a significant role in the interaction with host cells and with the immune system. OMV from a wide range of pathogenic bacteria have been found in various infected host tissues which suggests their contribution to pathogenesis. The immunomodulatory content of OMV (notably OM proteins, LPS, lipids) enables them to interact with host pattern recognition receptors to initiate the signaling cascades that lead to altered production of cytokines, chemokines, and antimicrobial peptides either to facilitate or limit inflammation (Kaparakis-Liaskos and Ferrero, 2015).

Production of OMV has previously been described in *F. novicida* (McCaig et al., 2013) and *F. tularensis* subsp. *tularensis* SchuS4 (Sampath et al., 2017) and a novel and unusual nanotubular shape was observed. Similar structures were also found in *F. noatunensis*, a related fish pathogen (Brudal et al., 2015). An increased OMV secretion together with enhanced biofilm formation and increased antibiotic resistance was observed in the *fupA*/*B* mutant of *F. tularensis* subsp. *holarctica* the live vaccine strain (Siebert et al., 2019). The protein composition of OMV was found to be related to the protein contents of the capsule-like complex (CLC) (Champion et al., 2018).

In the present study we demonstrate release of these nanotubular vesicles from *F. tularensis* subsp. *holarctica* strain FSC200, a virulent clinical isolate from a patient during the 1998 outbreak of ulceroglandular tularemia in central Sweden (Johansson et al., 2000). We have isolated OMV from bacteria cultivated at different conditions with the purpose to simulate various environments of *Francisella* life cycle. These included conditions mimicking the environment inside the mammalian host during infection: oxidative stress, low pH, and high temperature (42°C); and, in contrast, low temperature (25°C) that mimicked the external environment. The contribution of OMV to stress response and to host–pathogen interaction in *Francisella* is here demonstrated by differential OMV secretion under conditions simulating hostile environment of a mammalian host. The response was manifested by a several-fold increase in vesiculation rate at high temperature and low pH, both conditions characteristic of inflammation. A novel approach to the proteomic characterization of OMV is here presented in which a comparison of OMV protein cargo to membrane fraction is performed in order to find proteins significantly concentrated in OMV. Only these OMV-enriched proteins were further considered to be really involved in the OMV function and/or their exceptional structure. Further comprehensive proteomic characterization of OMV gave us an insight how the bacterium responds to the hostile environment of a mammalian host through the release of differentially loaded OMV.

## Materials and Methods

### Bacterial Strain and Growth Media

*Francisella tularensis* subsp. *holarctica* strain FSC200 (Johansson et al., 2000) was kindly provided by Åke Forsberg (Swedish Defence Research Agency, Umeå, Sweden). Stock bacteria were precultivated on McLeod agar supplemented with bovine hemoglobin and IsoVitalex (Becton Dickinson) at 37°C for 24 h. BHI (Becton Dickinson) was prepared according to manufacturer, pH was adjusted with HCl to 6.8 (unless otherwise stated), and it was sterile-filtered instead of autoclaving. For pH stress analysis, a scale of media was prepared as follows: pH 7.4 (unadjusted), 6.8, 6.3, 5.8, 5.3, 4.8, and 4.3.

### Determination of Growth Curves Under Stress Conditions

#### Temperature and pH Stress

Bacteria were inoculated into BHI and cultivated overnight at 37°C and 200 RPM. The overnight culture was pelleted (6000 × g, 15 min, 25°C) and diluted with fresh medium to OD_600_ 0.1. The growth curves at 37°C, 42°C, and at various pH (see Table 1 and above for the pH levels) were monitored in plate reader FLUOstar OPTIMA (BMG LabTech) for 42 h under constant shaking. OD was scanned every 10 min and it was normalized on 1 cm cuvette. OD at 25°C was monitored in a test-tube and measured at 0, 4, 16, 24, and 40 h.

**TABLE 1:** Cultivation conditions and stress treatments summary.

**Condition**		**Time of harvest (h)**	**OMV quantification**	**OMV isolation for TEM**	**SEM of bacteria**	**Proteomic comparison**
Untreated^a^	37°C, pH 6.8	16	Yes	Yes	Yes	Yes
High temperature	42°C	16	Yes	Yes	Yes	Yes
Low temperature	25°C	16/40	Yes/yes	Yes/no	Yes/no	Yes/no
pH	7.4	16	Yes	No	No	No
	6.3	16	Yes	No	No	No
	5.8	16	Yes	No	No	No
	5.3	16/40^b^	Yes/yes	Yes/no	Yes/no	Yes/no
	4.8	16	No	No	No	No
	4.3	16	No	No	No	No
Oxidative stress	5 mM H_2_O_2_	12 h of standard cultivation, 4 h of treatment	Yes	No	No	No
	10mM H_2_O_2_		Yes	Yes	Yes	Yes
	50mM H_2_O_2_		No^c^	No	No	No
Agar plate	McLeod	24	No	Yes	Yes	Yes

*^*a*^Untreated control, FSC200 cultivated in BHI pH 6.8, at 37°C for 16 h (late logarithmic phase); represents 100% of the vesiculation rate. ^*b*^At pH 5.3, the growth is delayed but it reaches untreated level at ca. 40 h of cultivation (see growth curves in Figure 4C). ^*c*^50 mM H_2_O_2_ had a considerable bactericidal effect (Figure 4A), large amount of dead cells distorted OMV quantification.*

#### Oxidative Stress

The overnight culture was pelleted as above, diluted with fresh medium to OD_600_ 0.1, and cultivated at 37°C for 12 h. Then hydrogen peroxide was added at final concentrations of 0, 5, 10, and 50 mM. The growth of bacteria was then monitored by OD reading and CFU plating every hour for next 4 h.

### OMV Quantification Under Stress Conditions

Bacteria for OMV quantification were cultivated in small scale (3 mL) in test-tubes under conditions described for growth curves. In the time of harvest a portion of the bacterial suspension was taken for serial dilution and CFU plating. The summary of cultivation conditions and harvest times for individual stress treatments is specified in Table 1. Bacteria were pelleted (10,000 × g, 15 min, 4°C) and the supernatants were sterile-filtered through syringe-driven filters (0.22 μm, PVDF). The quantity of OMV in the supernatants was assessed with the fluorescent dye SynaptoGreen^TM^ C4 (FM1-43, Biotium), similarly as described before (MacDonald and Kuehn, 2013). Culture supernatants were incubated with the dye at final concentration of 2.5 μg/mL for 10 min at 37°C in the dark. Fluorescence was measured using the FLUOstar OPTIMA plate reader at 485 (excitation) and 590 nm (emission). Acquired fluorescence was then normalized to CFU/mL at the time of vesicle harvest. Six to nine replicates were performed for each cultivation condition. Vesiculation rates were calculated as percent of the untreated cultivation. Ratios ± SD were estimated, one-way analysis of variance was used to evaluate the *p*-value, and *post hoc* test (FDR = 0.01) was employed to find the significant pairs against control. Statistical analysis was performed in Perseus software ver. 1.6.2.3 (Tyanova et al., 2016). In pH stress monitoring the pH of the culture media after bacteria harvest was checked using pH indicator strips (range 2.0–9.0, Macherey-Nagel) with the accuracy of 0.5.

### OMV Isolation

Outer membrane vesicles for TEM and MS analysis were prepared from large-scale cultivations (2–4 L). Overnight cultures were pelleted (6000 × *g*, 15 min, 25°C), diluted with fresh medium to OD_600_ 0.1, and cultivated as defined in Table 1. Bacteria were removed by centrifugation (10,000 × g, 20 min, 4°C), the supernatants were filtered through 0.22 μm PVDF filter and subsequently concentrated using Amicon^®^ Stirred Ultrafiltration Cell through the membrane of regenerated cellulose with nominal molecular weight cut-off of 100 kDa (both Millipore) to a final volume of ca. 20 mL. Concentrated supernatants were then pelleted (100,000 × *g*, 90 min, 4°C). The pellet was resuspended in 0.8 mL of 45% (w/v) OptiPrep (Sigma–Aldrich) in 10 mM HEPES/0.85% NaCl, pH 7.4 (HEPES buffer), and overlaid with OptiPrep gradient formed by: 40, 35, 30, 25, and 20% (0.8 mL each). The gradients were centrifuged at 100,000 × *g* for 16–20 h at 4°C in a swinging-bucket rotor. After centrifugation 12 0.4 mL fractions were carefully withdrawn from the top, a clearly visible opaque-white band was observed located in the upper part of the gradient (in fractions 1–3). Aliquots of the fractions were precipitated by 20% (w/v) trichloroacetic acid and subjected to SDS-PAGE. The proteins were visualized by Coomassie blue staining or electroblotted onto PVDF membrane, immunodetected with mouse monoclonal antibodies against LPS (FB11, Abcam), FopA, IglA, IglB, or IglC (all Moravian Biotechnology), and visualized by BM Chemiluminescence Blotting Substrate (Roche) on the iBright^TM^ FL1000 Imaging System (ThermoFisher Scientific). Fractions containing the visible white band were combined, diluted 8× with HEPES buffer, and centrifuged (100,000 × *g*, 90 min, 4°C). The supernatant was removed and the pellet was washed again (same conditions) to remove the residual OptiPrep. The final pellet was suspended in HEPES buffer and protein concentration was determined by Micro BCA^TM^ Protein Assay Kit (Pierce).

Outer membrane vesicles from agar plates grown bacteria were prepared as follows. Bacteria were grown on McLeod agar supplemented with bovine hemoglobin and IsoVitalex overnight. Bacteria were harvested into phosphate-buffered saline, washed gently by several aspirations in the pipette, and removed by centrifugation (10,000 × *g*, 20 min, 4°C). The washing step was repeated twice, supernatants were combined, and filtered through 0.22 μm PVDF filter. The procedure then followed as in the liquid culture OMV preparation.

### Transmission Electron Microscopy of OMV

Five microliters of OMV sample was applied onto glow-discharge activated formvar-/carbon-coated 300 Mesh copper grids (Benada and Pokorný, 1990). After 60 s of adsorption, the grids were negatively stained with 1% ammonium molybdate, pH 6.5. Alternatively, a mixture of 1% ammonium molybdate and 0.1% trehalose was used (Harris et al., 1995). The grids were examined in Philips CM100 Electron Microscope (Philips EO, now Thermo Fisher Scientific) at 80 kV. The digital images were recorded using MegaViewII or MegaViewIII slow scan CCD cameras (Sis GmbH; Olympus, now EMSIS GmbH). All digital images were processed in the AnalySis3.2 Pro software suite (Version Build 788, 2003) using standard modules (shading correction and digital contrast enhancement).

### Scanning Electron Microscopy of Whole Bacteria

Bacteria were cultivated in small-scale under conditions described in Table 1. A volume corresponding approximately to 2 × 10^9^ CFU was pelleted (6000 × *g*, 15 min, 4°C) and washed twice in 100 mM sodium cacodylate/5 g/L NaCl, pH 7.2. Bacteria were then fixed by 3% glutaraldehyde in the same buffer at RT for 1 h and then at 4°C overnight with slow rotation. Sterility of the fixed solution was checked by aliquot plating. The washed bacterial cells were then allowed to sediment overnight onto poly-L-lysine treated circular coverslips at 4°C. The coverslips with attached bacteria were post-fixed with 1% OsO_4_ for 1 h at room temperature and three times washed with ddH_2_O. Washed coverslips with the bacteria were dehydrated through an alcohol series (25, 50, 75, 90, 96, 100, and 100%) followed by absolute acetone and critical point dried from liquid CO_2_ in a K850 Critical Point Dryer (Quorum Technologies Ltd.). The dried samples were sputter coated with 3 nm of platinum in a Q150T Turbo-Pumped Sputter Coater (Quorum Technologies Ltd.). The final samples were examined in a FEI Nova NanoSEM 450 scanning electron microscope (Thermo Fisher Scientific) at 5 kV using CBS and TLD detectors.

### Preparation of Membrane-Enriched Fraction

Bacteria were cultivated as in the OMV isolation from the untreated control. Bacteria were washed twice by ice-cold phosphate-buffered saline, resuspended in 50 mM NH_4_HCO_3_ supplemented with protease inhibitors cocktail (complete EDTA-free, Roche Diagnostics), and disrupted by two passages in French pressure cell (Thermo IEC) at 1600 psi. The lysate was then treated with benzonase (Sigma–Aldrich) in a final concentration of 150 U/mL for 10 min on ice. Unbroken cells were removed by centrifugation (12,000 × *g*, 20 min, 4°C) and the supernatant was filter sterilized by syringe-driven 0.22 μm filter. The insoluble membrane fragments were pelleted at 100,000 × *g* for 30 min at 4°C, supernatant was discarded. Pellet was washed with fresh 50 mM NH_4_HCO_3_ supplemented with protease inhibitors and pelleted again. The final membrane pellet was suspended in 50 mM NH_4_HCO_3_ supplemented with protease inhibitors. Protein concentration was determined as above.

### Proteomic Analysis

For proteomic comparison of stress-derived OMV three large-scale cultivations were performed to evaluate three replicates from each condition: control (untreated), oxidative (10 mM H_2_O_2_ for 4 h), 42°C, 25°C, low pH (5.3), and McLeod agar plate. Each replicate was once digested and measured. For comparison of OMV vs. membrane fraction two large-scale cultivations were performed, each sample was split to two before digest to yield four final replicates. LC–MS/MS analysis was performed on Ultimate 3000 RSLCnano System (Dionex) coupled on-line through Nanospray Flex ion source with Q-Exactive mass spectrometer (Thermo Scientific). Data were processed by MaxQuant ver. 1.6.2.3 coupled with Andromeda search engine (Cox and Mann, 2008) and downstream proteomic analysis was performed in Perseus software ver. 1.6.1.1 (Tyanova et al., 2016). For detailed methods of proteomic analysis and data evaluation see Supplementary Material S1.

The MS proteomics data have been deposited to the ProteomeXchange Consortium via the PRIDE (Perez-Riverol et al., 2019) partner repository with the dataset identifier PXD013074.

## Results

### *F. tularensis* FSC200 OMV Have Tubular Shape

Bacteria were cultivated in the complex medium – BHI, which is known to support the bacterium to switch to the so-called host-adapted phenotype (Hazlett et al., 2008; Zarrella et al., 2011; Holland et al., 2017). This phenotype is characteristic of the production of longer O-antigen carbohydrate chains in LPS and higher molecular weight of capsular polysaccharides on the bacterial surface. The bacteria also elicit decreased proinflammatory response in macrophages and show an accelerated pathogenesis in mice (Zarrella et al., 2011). BHI also stimulates the bacterium in enhanced production of number of virulence factors together with the proteins from the LPS O-antigen gene cluster (Holland et al., 2017). The bacteria were grown to the early stationary phase for 16 h to reach an OD of ca. 0.6.

**FIGURE 1:**
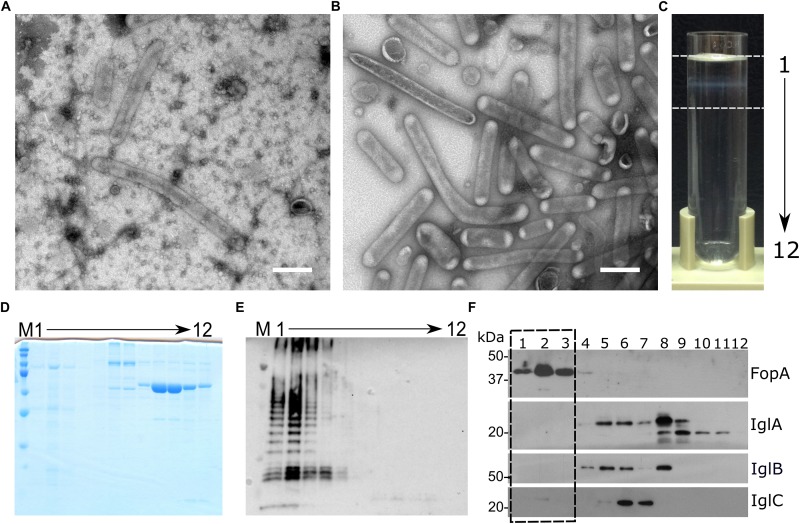
OMV isolation and purification. **(A)** TEM of pelleted culture filtrate and **(B)** OMV purified by density gradient; **(C)** density gradient separation – fractions 1–12 were collected from the top, white opaque band between the dashed lines corresponds to purified OMV; **(D)** SDS-PAGE of the density gradient fractions; **(E)** Western blot detection of LPS in the density gradient fractions; **(F)** Western blot detection of vesicular marker FopA and proteins IglA, IglB, and IglC in the density gradient fractions, dashed box highlights fractions 1–3 which were combined and taken as the OMV fraction for further analyses, corresponds to the part of the density gradient between the dashed lines in panel **(C)**. M, MW marker; 1–12, density gradient fractions collected from the top of the density gradient; **A** and **B** bars: 200 nm.

**FIGURE 2:**
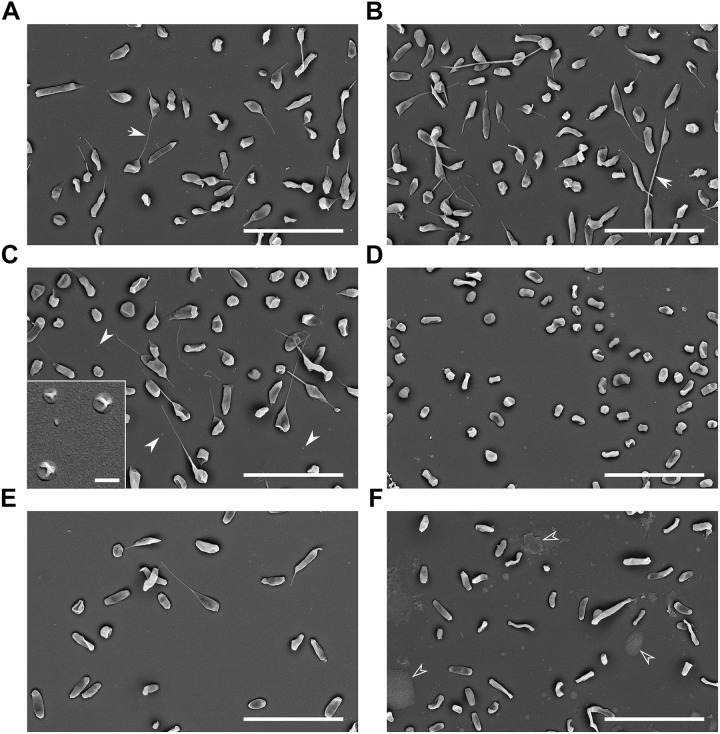
Scanning electron microscopy of bacteria cultured under various conditions. **(A)** Control: BHI, 37°C, pH 6.8, 16 h, arrows point to the tubular protrusions connecting adjacent cells; **(B)** oxidative stress: 10 mM H_2_O_2_; **(C)** 42°C, full arrowheads point to the small spherical vesicles on the background, inset: higher magnification of these small spherical vesicles; **(D)** 25°C; **(E)** pH 5.3; and **(F)** agar plate grown bacteria, empty arrowheads point to the unspecified material. Bars in main images: 5 μm; C-inset: 200 nm.

Outer membrane vesicles of tubular shape were isolated from FSC200 strain by culture medium ultrafiltration followed by high speed centrifugation and density gradient purification (Figure 1B). Simple pelleting of the secreted material led to a complex and rich mixture of spherical and tubular vesicles on the background of other extracellular material such as high molecular weight protein complexes and aggregates, chaperons, or pili (Figure 1A). Density gradient purification was thus performed to remove this material. OMV floated on the top of the density gradient as a clearly visible white opaque band (Figure 1C). Separation from the non-OMV-associated proteins was confirmed by SDS-PAGE and Coomassie staining of individual fractions (Figure 1D) and Western blot analysis by antibodies against LPS, FopA and IglA, IglB, and IglC (Figures 1E,F). LPS and the OmpA family protein FopA were here used as vesicular markers, while proteins from the FPI IglA, IglB, and IglC peaked in the medium density region of the gradient (Figure 1F) showing thus that they are probably not secreted *via* OMV. See in Supplementary Material S2 how the storage conditions influence the tubular shape and overall condition of the vesicles. SEM of the whole bacteria cultivated in BHI revealed the presence of membrane protrusions of the same tubular character as in the isolated OMV fractions (Figure 2). These tubules were most frequently budding directly from the bacterial surface, sometimes in two different directions from the same cell and often connecting two adjacent cells.

### Protein Characterization of OMV

Evaluation of the FSC200 OMV contents revealed quite a large group of 520 proteins. Many of them have previously been presented as OMV-associated in *F. novicida* (McCaig et al., 2013). The detailed list of all identified proteins is presented in Supplementary Table S1. To narrow the OMV protein pattern to proteins enriched in vesicles we compared the proteomic composition of isolated and purified OMV with the membrane fraction prepared from the whole cell lysate. We suppose that only the OMV-enriched proteins are the real effectors of OMV function(s) and/or contribute to their unusual shape. The proteomic comparison was performed using the algorithm of riBAQ (Krey et al., 2014), a label-free quantitative approach suitable for comparison of complex protein mixtures with large differences in protein abundancies. The enrichment coefficients were calculated as ratios of protein amounts in OMV over membrane. With the criteria described in Supplementary Material S1 the number of detected OMV-associated proteins decreased to 163, from which seven were more than 100× enriched in OMV, 48 were in the range of 10–100×, and 20 were found exclusively in OMV. The list of OMV most enriched proteins (enrichment factor higher than 20) is shown in Table 2. Full list of all OMV-enriched proteins with references regarding their role in virulence is in Supplementary Material S3 and the details from the proteomic analysis are in Supplementary Table S2.

**TABLE 2:** Proteins enriched in OMV in comparison with the membrane fraction, only proteins with enrichment factor >20 are shown.

**Protein name**	**Gene name**	**FTS locus tag**	**FTT locus tag**	**FTL locus tag**	**Enrichment^a^**
Outer membrane protein of unknown function		FTS_0008	FTT_1747	FTL_0009	424.2
Outer membrane protein OmpH	*ompH*	FTS_0538	FTT_1572c	FTL_0536	254.3
Hypothetical protein FTS_1462		FTS_1462	FTT_1334c	FTL_1494	246.4
Hypothetical protein FTS_1201		FTS_1201	FTT_0975	FTL_1225	126.9
Hypothetical protein FTS_1538		FTS_1538	FTT_0484	FTL_1579	126.5
Hypothetical protein FTS_0495		FTS_0495	FTT_0423	FTL_0493	102.0
Hypothetical protein FTS_0572		FTS_0572	FTT_1538c	FTL_0573	101.6
Hypothetical protein FTS_0814		FTS_0814	FTT_1137c	FTL_0822	73.0
Chitinase family 18 protein		FTS_1485	FTT_0715	FTL_1521	69.8
Hypothetical protein FTS_0402		FTS_0402	FTT_1303c	FTL_0411	60.9
Peroxidase/catalase	*katG*	FTS_1471	FTT_0721c	FTL_1504	56.7
Histidine acid phosphatase		FTS_0029	–	FTL_0031	56.5
Beta-lactamase class A	*bla*	FTS_0870	FTT_0611c	FTL_0879	48.9
Hypothetical protein FTS_1681		FTS_1681	FTT_0165c	FTL_1724	44.8
Hypothetical protein FTS_0755		FTS_0755	–	FTL_0755	38.4
Gamma-glutamyltranspeptidase	*ggt*	FTS_0764	FTT_1181c	FTL_0766	37.6
Rhodanese-like family protein		FTS_0824	FTT_1127	FTL_0834	35.5
Pyrrolidone carboxylylate peptidase	*pcp*	FTS_0203	FTT_0296	FTL_0207	35.5
Hypothetical protein FTS_1495		FTS_1495	FTT_0704	FTL_1532	35.3
Hypothetical protein FTS_1272		FTS_1272	FTT_0364c	FTL_1299	34.1
DNA topoisomerase I	*topA*	FTS_0417	FTT_0906c	FTL_0426	26.7
Protein-disulfide isomerase		FTS_1514	FTT_0507	FTL_1550	25.2
Exodeoxyribonuclease VII large subunit	*xseA*	FTS_0754	FTT_1190c	FTL_0754	24.5
Hypothetical protein FTS_0815		FTS_0815	FTT_1136c	FTL_0823	23.5
Hypothetical protein FTS_0974		FTS_0974	FTT_0540c	FTL_0994	23.4
Single-strand DNA-binding protein	*ssb*	FTS_0013	FTT_1752	FTL_0014	23.4
Outer membrane lipoprotein		FTS_1885	FTT_0211c	FTL_1939	21.9
Hypothetical protein FTS_0065		FTS_0065	FTT_1676	FTL_0073	20.4

*The entire list together with references regarding their role in virulence is in Supplementary Material S3. ^*a*^OMV/membrane enrichment coefficient.*

**FIGURE 3:**
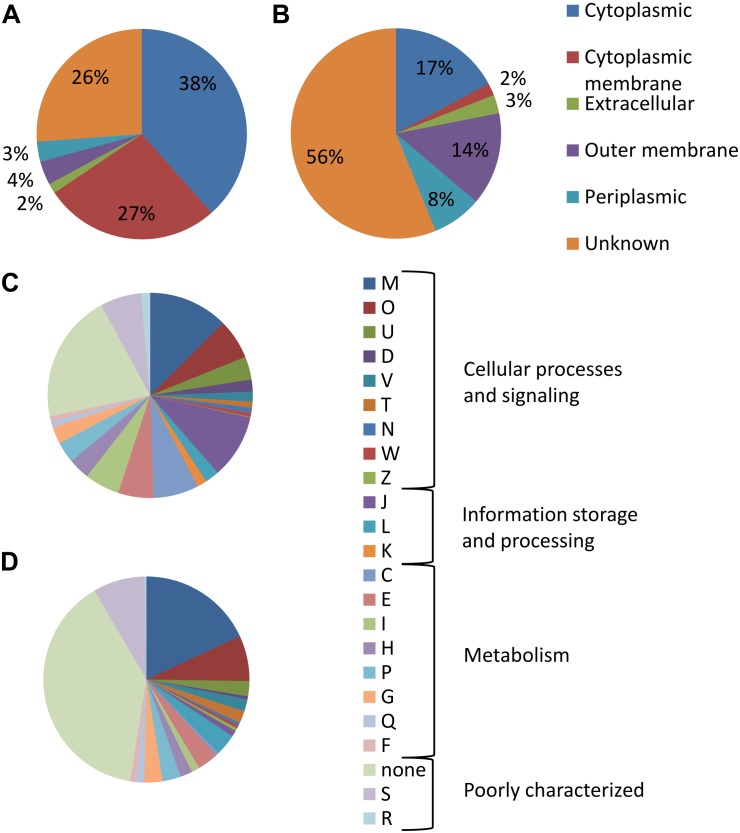
Categorization of detected proteins according to their cellular localization as predicted by PSORTb v.3.0.2: **(A)** in all identified OMV proteins and **(B)** in proteins enriched in OMV over membrane. Distribution of functional categories based on COG: **(C)** in all identified OMV proteins and **(D)** in proteins enriched in OMV. M (cell wall/membrane/envelope biogenesis), O (posttranslational modification, protein turnover, chaperones); U (intracellular trafficking, secretion, and vesicular transport), D (cell cycle control, cell division, chromosome partitioning), V (defense mechanisms), T (signal transduction mechanisms), N (cell motility), W (extracellular structures), Z (cytoskeleton), J (translation, ribosomal structure, and biogenesis), L (replication, recombination, and repair), K (transcription), C (energy production and conversion), E (amino acid transport and metabolism), I (lipid transport and metabolism), H (coenzyme transport and metabolism), P (inorganic ion transport and metabolism), G (carbohydrate transport and metabolism), Q (secondary metabolites biosynthesis, transport, and catabolism), F (nucleotide transport and metabolism), S (function unknown), and R (general function prediction only).

The enrichment resulted in remarkable changes in the distribution of functional categories as well as cellular localization when comparing enriched OMV proteins vs. all identified (Figure 3). Regarding cellular localization, the most visible changes were observed in cytoplasmic proteins and proteins from cytoplasmic membrane. The proportion of these proteins decreased in both cases; in cytoplasmic proteins from 38 to 17% and in cytoplasmic membrane proteins the decrease was even more prominent – from 27 to only 2%. In agreement with the OMV biogenesis mechanism, the portion of OM proteins increased from 4 to 14% and in periplasmic proteins from 3 to 8%. In the predicted functional categories, we have observed enrichment in “cell wall/membrane/envelope biogenesis” and in the general category of “poorly characterized proteins.” On contrary, an inconsiderable decrease was observed in the proportion of proteins from the general category of “information storage and processing,” especially those from “translation, ribosomal structure, and biogenesis” (from 10 to only 1%). This finding implicates that ribosomal proteins even though they are quite frequent in the vesicular samples don’t represent the real vesicular cargo but rather a contamination. Similarly, the following categories were decreased in OMV-enriched proteins: “energy production and conversion” (7 vs. 0.5%), “amino acid transport and metabolism” (6 vs. 3%), and “lipid transport and metabolism” (6 vs. 1%). In the non-enriched data, multiple subunits of the oxidative phosphorylation machinery were detected while none of them was significantly OMV enriched.

Fifteen members of the FPI were detected in the non-enriched OMV data, while only three of them passed the statistical criteria and with low enrichment coefficients: FTS_0101 (FTT_1335, PdpE, Hcp; enriched 3.9×), FTS_0111 (FTT_1346, IglE; enriched 3.7×), and FTS_0107 (FTT_1349, IglG; enriched 2.7×). We have observed that the FPI proteins with the greatest homology to the T6SS IglA, IglB, and IglC were not OMV-associated by the Western blot analysis of individual fractions from the OptiPrep density gradient (Figure 1F). While OMV were isolated from the top low density fractions, the IglA–C proteins peaked in the medium density part of the gradient.

Among the most enriched OMV proteins we have observed several major structural OM proteins of the OmpA and OmpH family, which are described to act as channel-forming transmembrane porins: OM protein of unknown function (OmpH-like, FTS_0008), OmpA family peptidoglycan-associated lipoprotein (pal, FTS_0334), OmpA family protein (FopA, FTS_1295), OmpA family protein (FTS_0323), as well as the OM protein OmpH (FTS_0538). The latter one is a Skp/OmpH molecular chaperone that interacts with unfolded proteins as they emerge in the periplasm from the Sec translocation machinery. Similarly, the proteins involved in the Tol-PAL system which is required for the bacterial OM integrity were enriched in OMV: the group A colicin translocation tolB protein (TolB, FTS_0332), hypothetical protein FTS_1076 (type I secretion OM protein, TolC family), OM protein tolC precursor (TolC, FTS_1817), TPR repeat-containing protein (a putative tol-pal system protein YbgF, FTS_0201), and OM efflux protein (SilC–TolC ortholog, FTS_0687).

Regarding the major OM proteins mentioned above, it should be noted that also the components of Bam-complex (β-barrel assembly machinery) were highly enriched in OMV: OM protein of unknown function (BamA, FTS_0537), hypothetical protein FTS_1681 (BamB), competence lipoprotein ComL (FTS_0701, putative BamD), and OM lipoprotein (OmlA, FTS_0061, putative BamE). The Bam complex is responsible for the assembly of other β-barrel proteins into the OM of virtually all Gram-negative bacteria and homologs are found also in mitochondria and chloroplasts (Malinverni and Silhavy, 2011).

Other proteins with previously described relation to virulence and/or immunoreactivity were found to be concentrated in OMV. Examples concern the lipoprotein of unknown function (LpnA, FTS_0412, 17 kDa lipoprotein TUL4 precursor) and hypothetical protein FTS_0415 (LpnB, a putative TUL4), LpnA is the immunodominant lipoprotein of *F. tularensis* and a known TLR2 agonists (Sjöstedt et al., 1992; Forestal et al., 2008; Thakran et al., 2008). Furthermore, histidine acid phosphatase (FTS_0029) was highly OMV enriched together with two other acid phosphatases (histidine acid phosphatase FTS_0996 and hypothetical protein FTS_1178). Acid phosphatases hydrolyze phosphate esters, optimally at low pH, and they are suspected to be involved in the virulence by inhibiting reactive oxygen species production in host cells. Disruption of all four acid phosphatases in SchuS4 strain disabled it from the phagosomal escape (Mohapatra et al., 2013). Three proteins were here significantly OMV-enriched that were previously found up-regulated after infection in macrophages: hypothetical protein FTS_1538, peptide methionine sulfoxide reductase (MsrA, FTS_1906), and ATP-dependent Clp protease proteolytic subunit (ClpP, FTS_0883) (Pávková et al., 2013). Two chitinases (ChiA, FTS_1485 and ChiB, FTS_0083) were here found among the most OMV-enriched proteins together with the hypothetical protein FTS_1749, another putative chitinase class II group protein.

### Stress Induced OMV Release

We exposed the bacteria to several cultivation conditions that induced various growth stresses to simulate the diverse conditions of *F. tularensis* life cycle. These included oxidative stress, low pH, high temperature (42°C), low temperature (25°C), and as an alternative to liquid media cultivation we have also isolated OMV from agar plate-grown bacteria.

**FIGURE 4:**
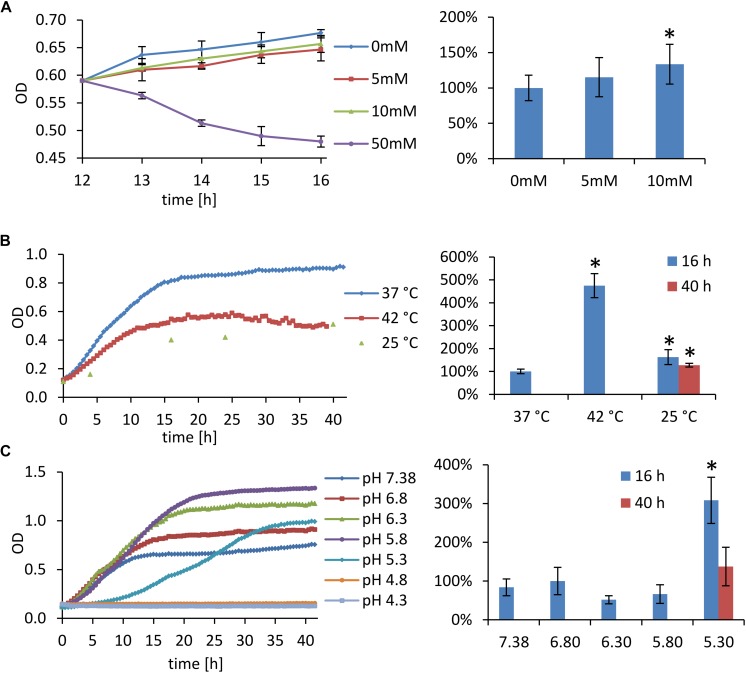
Stress cultivation growth curves (left) and relative vesiculation rates (right). Fluorescence of the culture media was normalized to CFU/mL and expressed as percent of the control cultivation. **(A)** Oxidative stress: bacteria were grown for 12 h under standard cultivation conditions, then they were treated with 5–50 mM H_2_O_2_ (50 mM H_2_O_2_ had a considerable bactericidal effect, large amount of dead cells distorted OMV quantification); **(B)** high and low temperature stress; and **(C)** cultivation at different pH. Results show ratios ±SD from six to nine independent experiments; ^∗^ significant (*p* < 0.01) against control cultivation.

**FIGURE 5:**
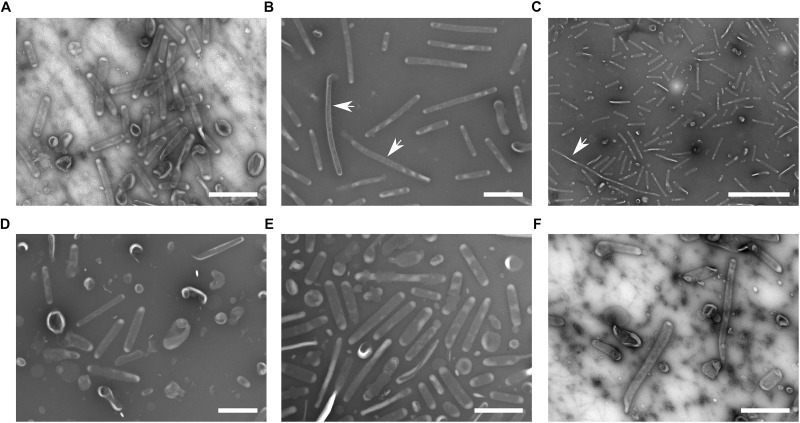
Transmission electron microscopy of vesicles isolated from different cultivation conditions. **(A)** Oxidative stress caused by 10 mM H_2_O_2_; **(B,C)** 42°C; **(D)** 25°C; **(E)** pH 5.3; and **(F)** vesicles from agar plate grown bacteria. Arrows point to the extremely long nanotubes characteristic for high temperature cultivation. Bars **A,B** and **D–F**: 0.5 μm; and **C**: 2 μm.

Outer membrane vesicles for relative quantification of the vesiculation rate were not isolated and purified from the culture supernatants but they were stained directly in the bacterium-free culture media. For this purpose the fluorescent membrane probe FM1-43 was employed. This dye has negligible fluorescence in aqueous solutions but it has high affinity to lipid membranes and upon insertion into the membrane its fluorescence increases. The fluorescence of the solution is thus an indirect measure of membranes in the medium. Fluorescence of culture media was normalized to CFU/mL at the time of harvest and expressed as percent of the untreated cultivation. Table 1 summarizes cultivation conditions used in the study and Figure 4 shows the respective growth curves and relative vesiculation rates under different conditions. The conditions that led to the greatest changes in vesiculation rates were further used to study the OMV morphology by TEM (Figure 5), morphology of the bacteria by SEM (Figure 2), and proteomic comparison.

#### Oxidative Stress

To induce oxidative stress hydrogen peroxide was here added to exponentially growing bacteria after 12 h of standard cultivation in the final concentrations of 5, 10, and 50 mM, respectively. The cultivation then continued for next 4 h. As resulted from the growth curves (Figure 4A), 5 and 10 mM H_2_O_2_ had negligible influence on the growth, while 50 mM H_2_O_2_ was bactericidal. The concentrations of 5 and 10 mM had also quite low effect on the vesiculation rate, which was only raised by 15 and 34%, respectively. For further analyses only the 10 mM concentration was used and it revealed similar OMV morphology (Figure 5A) as in the standard cultivation conditions (shown in Figure 1B). No visible effect was also observed on the morphology of treated bacteria (Figures 2A,B).

#### High and Low Temperature Cultivation

The growth of FSC200 at 42°C was here reduced to ca. one half of the control cultivation (Figure 4B, left); nevertheless, the viability of bacteria wasn’t affected and a considerable increase in the secreted membrane material was observed, the rate being on 475% in comparison with 37°C cultivation (Figure 4B, right). Moreover, TEM of the vesicles here isolated revealed a great number of extremely long nanotubes (Figures 5B,C). On the SEM images of the 42°C treated bacteria (Figure 2C) the protrusions were slightly longer than in control and on the background there were numerous spherical membrane vesicles (Figure 2C-inset).

At 25°C the growth was slightly lower than at 42°C and the vesiculation rate was only 63% above the control after 16 h of cultivation (Figure 4B). In contrary to the high temperature-derived vesicles, these appeared to be more “damaged” with a relatively lower proportion of the nanotubes and higher number of spherical vesicles among which a larger variety of diameters was observed than in the control samples (Figure 5D). SEM images of these bacteria show no tubular protrusions and a little amount of spherical vesicles, the size of bacteria was considerably smaller (Figure 2D).

#### Low pH Stress

Culture media were here adjusted to pH ranging from 7.4 (the non-adjusted BHI medium) to 4.3. According to the growth curves (Figure 4C), pH optimum for FSC200 growth ranged between 5.8 and 6.3, but vesiculation rate in these media was comparable or even slightly lower than in control (pH 6.8). At extremely low pH (4.8 and lower) the bacteria didn’t grow at all and vesiculation rate wasn’t determined. The vesiculation was most enhanced at pH 5.3, reaching as much as 308% of the control cultivation at the same harvest time. The growth at pH 5.3 was considerably retarded in the beginning, but after ca. 35 h it reached the same OD as control. The pH value of media after the harvest of bacteria was checked and except for the extremely low values, the pH raised gradually during cultivation to ca. 7.0–7.5. In the case of starting pH 5.3 the value raised to 6.0 after 16 h and it reached 7.0 after 40 h (see Supplementary Material S4). The morphology of isolated vesicles resembled to the control sample (Figure 5E), morphology of bacteria showed lower amount of protrusions than control (Figure 2E).

#### OMV From Agar Plate Cultivated Bacteria

Isolation and purification of OMV in good quality, quantity, and satisfactory reproducibility presents an inconsiderable challenge. The yields of OMV isolated and purified from FSC200 strain under the described standard cultivation conditions (BHI medium, pH 6.8, 37°C, late exponential phase of growth) were quite low, ranging usually around 20–30 μg of protein per 1 L of the bacterial suspension. For this reason we have tested the possibility to avoid the large volume cultivations by isolating OMV from agar plate cultivated bacteria. The bacteria were harvested from the McLeod agar plates after an overnight cultivation at 37°C and extracellular material was washed three times into PBS. The yields were good and the preparation was much faster and easier; however, the vesicles differed in their morphology from the liquid media cultivation (Figure 5F) resembling most to those isolated from 25°C cultivation with even more damaged appearance. Similarly, the whole bacteria as visualized by SEM expressed higher variability in size and shape and a considerable amount of unspecified intercellular material was observed on the background (Figure 2F), possibly a material resembling the CLC as described in Bandara et al. (2011) and Champion et al. (2018). Moreover, as the proteomic comparison indicated, the composition of such sample differed too much from the control probably due to material from dead cells. For these reasons we conclude that agar cultivation is not suitable for further OMV research.

### Protein Cargo of OMV in Different Stress Conditions

The proteomic comparison was performed similarly to the enrichment study described above by label-free quantitative approach with the algorithm of riBAQ. Six samples were included in the comparison: untreated control, oxidative stress, low pH of 5.3, high temperature of 42°C, low temperature of 25°C, and agar plate cultivation; the conditions and harvest times are summarized in Table 1. Two approaches were applied on the same data. First, the whole dataset of all quantified proteins was evaluated to see the overall quality of the data and comparability between the samples. Multiple sample test (ANOVA) was used to compare the differences among all cultivation conditions. Hierarchical clustering was employed to find proteins with similar responses to the studied different conditions and Fisher exact test was applied on the resulting clusters to find significantly enriched categories in protein annotations as metabolic pathways or subcellular localizations. In the second approach the ANOVA significant proteins were filtered on proteins previously in this study found to be OMV-associated.

From 843 proteins identified from the whole dataset 467 were ANOVA significant, see Supplementary Table S3 for the detailed proteomic comparison. The PCA revealed a good clustering of the cultivation conditions according to their similarity (Supplementary Material S5). Oxidative stress appeared to be the most similar to untreated control. High temperature clustered with low pH cultivation which is in agreement with the fact that both these two conditions are characteristic for the hostile environment of infected mammalian host during inflammation. On the other hand, cultivation at 25°C and on agar plate appeared to induce the most changes in OMV composition, the observations corresponding to the most damaged view of isolated OMV.

This part of the study was focused on the identification of the clusters of proteins with patterns characteristic for different cultivation conditions. The obtained clusters are summarized in Supplementary Material S6 (Supplementary Figure 6-1 and Supplementary Table 6-1). The proteins that were significantly more abundant at 25°C and on agar and at the same time unchanged in all other conditions (cluster 435) were statistically enriched in proteins with predicted cytoplasmic localization. Similarly, proteins with the opposite pattern (decreased on agar and at 25°C while unchanged in all other conditions, clusters 414 and 327) were enriched in proteins with predicted unknown and periplasmic localization and in proteins designed in this study as OMV-associated, too. These results suggest that OMV from agar plates and from 25°C cultivation were probably significantly contaminated by material from dead or non-proliferating cells. Proteins in cluster no. 406 were unchanged at 25°C and on agar while significantly down-regulated at high temperature, oxidative as well as pH stress. This cluster was enriched in proteins from the KEGG pathways: RNA polymerase, pyrimidine metabolism, and purine metabolism, and in proteins with Gene Ontology term GO:0006807 (nitrogen compound metabolism). Two small clusters were observed with the same change direction in all studied conditions in comparison to control. Proteins in cluster no. 157 were raised in all conditions over control and contained putative ABC transporter ATP-binding protein (FTS_1893), isocitrate dehydrogenase (FTS_0587) and acetyl-CoA carboxylase, and biotin carboxylase subunit (FTS_1551). On the other hand, proteins from cluster no. 430, which was decreased in all conditions over control, included two FPI proteins (FTS_0099, IglC and FTS_0107, IglG) and a putative glycosyltransferase that occurs in three copies in the FSC200 chromosome (hypothetical protein FTS_1842, FTS_1289, FTS_0261).

A relatively high number of proteins that participate on LPS biosynthesis were among the ANOVA-significant group obtained in this study. The *wbt* locus was here quantified on 14 out of 15 proteins and 11 of them were ANOVA-significant, see Supplementary Material S6 (Supplementary Figure 6-2A and Supplementary Table 6-2). Genes from this cluster are involved in O-antigen and capsule biosynthesis (Raynaud et al., 2007; Apicella et al., 2010). Two distinct patterns of proteins abundances in different cultivation conditions were observed. The first group (WbtC, WbtD, WbtE, WbtG, WbtH, WbtI, and WbtK) was unchanged in oxidative stress, much decreased in low pH and in 42°C, and slightly decreased at 25°C. These proteins were described to participate in the biosynthesis of the first three sugar moieties of the four-sugar O-antigen subunit and their glycosidic binding (Prior et al., 2003). Proteins in the second group (WbtB, Wzy, and Wzx) were increased in oxidative stress, at 25°C and on agar plate, and decreased in low pH and in 42°C. WbtB is proposed to initiate O-antigen biosynthesis by adding the first sugar moiety to undecaprenyl phosphate, Wzy is an O-antigen polymerase, and Wzx an O-antigen flippase (Prior et al., 2003). Finally, protein WbtJ was only slightly elevated at high temperature, oxidative and pH stress while extremely elevated at 25°C and on agar. This protein is a formyltransferase responsible for formylation of the fourth sugar moiety (Prior et al., 2003).

Lipid A (endotoxin) biosynthetic pathway was also strongly represented in our results. From 10 proteins that take part in the biosynthesis of lipid A and joining it with KDO 9 were here quantified and 5 were ANOVA-significant, see Supplementary Figure 6-2B and Supplementary Table 6-2. These proteins were decreased in low pH and in 42°C, and increased at 25°C and on agar plate. They were unchanged in oxidative stress except for LPS fatty acid acyltransferase (FTS_0176, HtrB), which was there increased.

A similar pattern to that described above (decreased in low pH and in 42°C, and increased or not changed at 25°C and on agar plate) was also observed in proteins involved in the LPS export system except for the organic solvent tolerance protein, OstA (OstA1 or LptD, FTS_1557) that was unchanged at high temperature, oxidative and pH stress but decreased at 25°C and on agar plate (Supplementary Figure 6-2C and Supplementary Table 6-2). The pattern was also followed by proteins from phospholipid biosynthesis and transport pathways (Supplementary Figure 6-2D and Supplementary Table 6-2): three phospholipid acyltransferases (PlsC, FTS_0079; PlsC, FTS_1865; and PlsX, FTS_1113) and four phospholipid ABC transporters (MlaD, FTS_0516; MlaF, FTS_0518; MlaD, FTS_0696; and MlaE, FTS_0698). The fifth phospholipid ABC transporter, a membrane protein of unknown function (ttg2 or MlaC, FTS_0515, a toluene tolerance protein) was unchanged in all the stresses but it was decreased at 25°C and on agar plate.

We also report changes in the metabolic pathways of fatty acids. Twelve proteins of this pathway were ANOVA-significant (Supplementary Figures 6-2E,F and Supplementary Table 6-2). Most of them were unchanged at high temperature, oxidative and pH stress but increased at 25°C and on agar plate. Three proteins of the fatty acid synthase complex (FabG, FTS_1110; FabI, FTS_1414; and CaiC, FTS_0691) were decreased only at low pH. FabG was the only one OMV-enriched.

In further comparison of the influence of different cultivation conditions on OMV protein content the data were reduced to OMV-enriched proteins only, which revealed 91 ANOVA-significant proteins (highlighted in Supplementary Table S3). Hierarchical clustering on this dataset revealed two large clusters of proteins that were all similarly decreased at 25°C and on agar plate and increased at high temperature, oxidative as well as at pH stress – clusters nos. 56 and 57 (Supplementary Figure 6-3). The two clusters together contained 35 proteins (Supplementary Table 6-3), which was more than one-third of all significant proteins. These proteins are thus expected to be preferentially packed into OMV during infection. An inconsiderable portion of them has previously been mentioned in publications to be connected to virulence or strong immunostimulators (Kilmury and Twine, 2011; Chandler et al., 2015). Most of them are hypothetical with no known or proposed function; few exceptions are here referred. Peroxidase/catalase (FTS_1471, KatG) is connected to resistance of *Francisella* against reactive oxygen species (Lindgren et al., 2007), it is secreted into culture media (Konecna et al., 2010) and into macrophages during infection where it restricts macrophage signaling and cytokine production (Melillo et al., 2010). One of the above mentioned acid phosphatases (FTS_0029) was found in this cluster too, as well as all three chitinases discussed above in connection with biofilm formation (ChiA, FTS_1485; ChiB, FTS_0083; and FTS_1749). The first one was previously found to be highly up-regulated *in vivo* in mice (Twine et al., 2006), while both ChiA and ChiB were found to have anti-biofilm formation properties in *F. novicida* (Chung et al., 2014). Peptide methionine sulfoxide reductase (FTS_1906, MsrA) and methionine sulfoxide reductase B (FTS_0370, MsrB) are repair enzymes for proteins that have been inactivated by oxidation and were both described to be up-regulated in macrophages in the early stages post-infection (Wehrly et al., 2009; Pávková et al., 2013).

For hypothetical protein FTT_1538c (a homolog of FTS_0572 in *F. tularensis* subsp. *tularensis* SchuS4 strain) the interactions with host cell proteins AP3M1 and WDR48 were previously discovered which strongly links this protein to biological processes involved in intracellular fate of the bacterium (Wallqvist et al., 2015). AP3M1 is involved in the maturation of endosomes to late endosomes and lysosomes and interfering with this molecule could alter endosome maturation or endosome–lysosome fusion. WDR48 regulates deubiquitinating complexes and interaction with it might provide a mechanism to interfere with targeted destruction of pathogen proteins. FTS_0572 thus probably contributes to intracellular survival by interfering with vesicular trafficking and contributes to phagosomal escape (Wallqvist et al., 2015). Together with its 100× enrichment in OMV in comparison to membrane makes it a very interesting OMV-associated protein. Hypothetical protein FTS_0163 is a putative regulator of chromosome condensation, but according to Rohmer et al. (2007) it contains an α-tubulin suppressor domain and may thus interact with the cytoskeleton of the host cell which also connects the protein with intracellular trafficking.

## Discussion

Release of OMV plays an important role in the physiology and/or pathogenesis in all Gram-negative bacteria; nevertheless, the nanotubular shape of OMV secreted by *Francisella* spp. is quite unique among other bacteria. The vesicles here obtained from *F. tularensis* subsp. *holarctica* strain FSC200 were comparable in size and shape to those that were previously documented in *F. novicida* and *F. tularensis* subsp. *tularensis* SchuS4 (McCaig et al., 2013; Sampath et al., 2017). Similarly, the nanotubular protrusions as well as membranous bridges between adjacent bacteria were described in *F. tularensis* LVS (Gil et al., 2004).

The proteomic analysis of OMV fraction revealed a huge number of proteins. The group contained a considerable number of previously described virulence factors as well as proteins with known immunostimulatory potential (Janovská et al., 2007; Kilmury and Twine, 2011; Chandler et al., 2015; Straskova et al., 2015). Nevertheless, the number of detected proteins seems to be excessively high regarding that it forms approximately one-third of the *F. tularensis* proteome database. According to the mechanism of OMV biogenesis their protein cargo should contain preferentially OM and periplasmic proteins; however, the relative amounts of individual proteins differ a lot between OMV and isolated OM or periplasmic fractions. Furthermore, an inconsiderable portion of proteins with predicted cytoplasmic or inner membrane localization are being found in vesicles as well. These findings indicate that there is a yet unknown mechanism of specific protein enrichment and/or exclusion during the process of OMV formation (Haurat et al., 2011, 2015; Bonnington and Kuehn, 2014). From this point of view, the OMV biogenesis doesn’t only represent a passive membrane shedding with random diffusion of envelope contents, but it is an active process. In this way, bacterium can flexibly respond to actual environmental changes.

For these reasons we searched for proteins that are effectively enriched in OMV in comparison to the membrane fraction. Even though many proteins have previously been attributed to OMV in *Francisella* (Pierson et al., 2011; McCaig et al., 2013; Sampath et al., 2017; Champion et al., 2018), we have shown here that many of them do not reach OMV for a particular purpose but rather because they reside on the membrane or in periplasm from which OMV originate. For example, the proteins from the oxidative phosphorylation pathway or, even more relevant, the FPI proteins, represent a notable proof of this phenomenon. Some of the FPI proteins are structural and/or functional constituents of T6SS (Bröms et al., 2012; Clemens et al., 2015; Spidlova and Stulik, 2017); as such they are probably not released by the vesicular mechanism. Some of them are expressed and secreted (or mechanically shed) in quite high amounts so they can be found in the vesicular fractions as well (McCaig et al., 2013). Recently also the localization of IglE in OM was confirmed (Bröms et al., 2016), which further explains its presence in OMV. These facts suggest that these proteins reach OMV by random diffusion in the membrane.

On the other hand, the most OMV-enriched proteins were from the groups of the channel-forming transmembrane porins, peptidoglycan-associated glycoproteins, and proteins from the Tol-PAL system. Many of these proteins are known to be highly immunogenic in *F. tularensis* (Kilmury and Twine, 2011). Moreover, proteins from this group also generally serve as receptors of bacteriophages, targets of bacteriocins, or other natural antibiotics and in the case of pathogenic bacteria serve also as targets for human antimicrobial peptides and antibodies. From this point of view the secretion of OMV helps to defend the bacterium during its extracellular phase because they function as a decoy to those OM-acting agents and can reduce their concentration to the sub-lethal level (Manning and Kuehn, 2011).

From a different point of view OMV secretion not only relates to virulence but also enables bacteria to survive and prosper during their environmental phase. The chitinases here reported as highly OMV-enriched present an example of such environmental adaptation. Chitinases A and B enable *Francisella* to persist on chitin as a sole carbon source (Margolis et al., 2010), they are essential for biofilm formation (van Hoek, 2013) and in *F. novicida* they are negative regulators of biofilm formation (Chung et al., 2014).

We further described the contribution of OMV to stress response in *Francisella*. Differences in OMV secretion under various cultivation conditions were observed in their amount, morphology, and also in their protein cargo. The conditions were designed to simulate either the environment inside a homeothermic host during infection or the external environment. Oxidative stress caused by sub-lethal concentration of H_2_O_2_ was performed to mimic treatment of the bacteria by the reactive oxygen species, which represent one of the host-employed microbicidal strategies during *F. tularensis* infection. A straight correlation has previously been described between *in vitro* resistance to H_2_O_2_ and the virulence of *F. tularensis* strains: the subsp. *tularensis* SchuS4 strain was more resistant than subsp. *holarctica* FSC200 and the attenuated LVS strain was the least resistant (Lindgren et al., 2007, 2011). Nevertheless, here the response to H_2_O_2_ treatment was negligible in vesiculation rate as well as in the protein load. These findings suggest that either oxidative stress alone is not a strong inducer of vesiculation and that H_2_O_2_ detoxification doesn’t rely on OMV release, similarly as stated earlier in *F. novicida* (Sampath et al., 2017), or FSC200 response is too rapid and efficient to be captured by the used methods.

On the other hand, elevated temperature as well as low pH raised the amount of membranous material in the culture supernatants similarly by several fold and also the protein changes were also comparable. Both these conditions were characterized also by lower bacterial proliferation as assessed by the growth curves. Heat stress associated with the transfer of the bacterium into the mammalian host was previously described to induce the production of virulence factors in *Francisella* (Horzempa et al., 2008; Lenco et al., 2009) and to increase the *in vitro* and *in vivo* virulence of the bacterium (Bhatnagar et al., 1995). Hyper-vesiculation was also described in other bacteria as a general response to high temperature stress through which bacteria avoid accumulation of misfolded proteins in their envelope (McBroom and Kuehn, 2007). The presence of extremely long nanotubes in higher temperature cultivation suggests that the stability of the nanotubes was enhanced so that they were more rigid and more resistant to mechanical damage during cultivation and sample processing. In *F. novicida* an alteration in the lipid A structure was described upon a temperature shift from environmental to mammalian temperature and the authors suggested that LPS/lipid A modifications resulting in alterations of membrane fluidity, as well as integrity, may represent a general paradigm for bacterial membrane adaptation and virulence-state adaptation (Shaffer et al., 2007; Li et al., 2012). Nevertheless, we also observed a significant amount of spherical membrane vesicles present on the background of the heat stressed bacteria as shown by SEM. This observation explains the several fold increase in the vesiculation rate rather than the presence of longer nanotubes or higher number of them.

Regarding the low pH stress, in *F. tularensis* the maturation of bacteria containing vacuole is arrested and it does not fuse with lysosome but, on the contrary, the bacteria disintegrate the vacuole membrane by a not yet elucidated mechanism, escape to the cytosol, and multiply there. The question of acidification of the bacteria containing vacuole remains disputable but at least transient acidification cannot be ruled out (Clemens et al., 2004, 2009). It has been described previously that when *F. tularensis* is cultured in acidified medium, the pH of the medium increases (Chamberlain, 1965), reportedly due to the generation of ammonia (Traub et al., 1955; Larsson et al., 2005). The high vesiculation rate at low pH together with the ability of *Francisella* to adjust the pH of its medium to a more favorable level suggests that vesiculation is somehow related to avoiding the acid pH stress.

Significant protein changes observed in the non-OMV-enriched data indicate alterations of the enzymatic pathways involved in the biosynthesis and metabolism of the envelope components: O-antigen, lipid A, phospholipids, and fatty acids. Most proteins that participate on the LPS biosynthesis expressed an opposite response to high temperature, oxidative and low pH stress when compared to low temperature and agar cultivation. O-antigen modification of LPS as well as the polysaccharide capsule play an important role in the *Francisella* virulence strategy of avoiding the immune system recognition (Rowe and Huntley, 2015) and O-antigen remodeling under different cultivation conditions is highly probable. Similarly, it has been described previously that *Francisella* and other bacteria with environmental and mammalian reservoirs can modify their lipid A in response to environmental stimuli, especially temperature (Li et al., 2012; Powell et al., 2012). Furthermore, the presence, composition, and amount of the CLC which is a complex mixture of high molecular weight polysaccharides with proteins, and glycoproteins, also relates to the cultivation conditions (Champion et al., 2018).

On the other hand, only three of the reported proteins involved in any stage of the LPS biosynthesis were found to be OMV-enriched and their function is thus expected to be directly associated to OMV. One of them was WbtE (FTS_0595), which takes part in the biosynthesis of O-antigen. Two other of these proteins were unchanged at high temperature, oxidative, and low pH stress but they were strongly decreased at low temperature and on agar: OstA1 (or LptD, FTS_1557) and ttg2 (or MlaC, FTS_0515). The first one takes part in the transport and assembly of LPS on the outer leaflet of OM and the second one maintains lipid asymmetry in the OM by retrograde trafficking of phospholipids from the OM to the inner membrane and thus prevents phospholipid accumulation at the cell surface. These results suggest that the modulation of cell surface as well as modulation of OMV surface is activated in response to hostile environment. Likewise, the similarity in the responses of the LPS and phospholipid biosynthetic pathways makes sense because these pathways in Gram-negative bacteria are synchronized to achieve a proper balance in membrane composition (Emiola et al., 2016). The reported changes in the metabolic pathways of fatty acids are also in close connection to the above discussed LPS and phospholipid biosynthesis changes as well as to the rigidity of the bacterial/OMV membrane.

Regarding their biogenesis, the protein composition of OMV highly reflects the composition of bacterial OM plus the periplasmic compartments. Nevertheless, the mechanism of selection of proteins to be preferentially secreted *via* OMV is still unknown. Having this in mind, it is questionable whether the observed proteome changes of OMV isolated from different cultivation conditions correspond to the overall regulations in the whole cellular proteome or they really reflect the stress-induced directing of particular proteins into OMV. The reduction of the studied proteins to OMV-enriched gave us a more specific insight on how the bacterium responses to the hostile environment of a mammalian host through the release of differentially loaded OMV. The proteins selectively packed into OMV mostly involved hypothetical proteins with unknown function but some previously described virulence factors connected to the unique intracellular fate of *Francisella* were found, as well. Together with the above discussed modulations of bacterial membrane and OMV surface structures in response to either host or environmental compartments, the results also show the possibility that proteins may be selectively directed to OMV in response to stressful host-like conditions and that some of these proteins thus might interfere with intracellular trafficking mechanisms.

Packaging of secreted proteins into OMV has many advantages including their enhanced stability to proteolysis or avoiding recognition by immune system. Beyond that, we can also expect interaction of the membrane of OMV with endosomal membranes and misguiding of the classical endosomal pathway. Such membrane-to-membrane interactions on the intracellular level could have some relation with endosomal membrane disruption and *Francisella* escape to the cytosol. Further studies directed on the OMV fate during infection are required to prove that.

## Data Availability Statement

The datasets generated for this study can be found in the ProteomeXchange, PXD013074.

## Author Contributions

JK and JS conceived and designed the study. JK, IP, LH, and JB performed the experiments. OB and OK performed and interpreted the electron microscopy. JK performed the MS analyses and bioinformatic analysis, and wrote the manuscript. JS and IP edited the manuscript.

## Conflict of Interest

The authors declare that the research was conducted in the absence of any commercial or financial relationships that could be construed as a potential conflict of interest.

## Supplementary Material

TABLE S1 List of all proteins identified in OMV.

TABLE S2 List of proteins that were OMV enriched in comparison with membrane fraction.

TABLE S3 Proteomic comparison of OMV from different cultivation conditions.

MATERIAL S1 Detailed methods of proteomic analysis and proteomic data evaluation.

MATERIAL S2 Notes to the OMV storage.

MATERIAL S3 Full list of all OMV-enriched proteins with references regarding their role in virulence.

MATERIAL S4 pH of BHI medium before and after cultivation of *F. tularensis* subsp. *holarctica* strain FSC200.

MATERIAL S5 Exploratory analysis of the proteomic data – comparison of OMV isolated from different cultivation conditions.

MATERIAL S6 Hierarchical clustering of the proteomic data – comparison of OMV isolated from different cultivation conditions.

## Abbreviations

BHI: brain heart infusion
CFU: colony-forming unit
FPI: *Francisella* pathogenicity island
LC–MS/MS: liquid chromatography coupled with tandem mass spectrometry
LPS: lipopolysaccharide
MS: mass spectrometry
OM: outer membrane
OMV: outer membrane vesicles
PCA: principal component analysis
PVDF: polyvinylidene difluoride
riBAQ: relative intensity-based absolute quantification
T6SS: type six secretion system
SEM: scanning electron microscopy
TEM: transmission electron microscopy.
