# Supplementary material for: Francisella tularensis subsp. holarctica Releases Differentially Loaded Outer Membrane Vesicles Under Various Stress Conditions
Source: Front Microbiol. 2019 Oct 10;10:2304. doi: 10.3389/fmicb.2019.02304 (PMC6795709; doi:10.3389/fmicb.2019.02304)
Supplement: MATERIAL S1 — Detailed methods of proteomic analysis and proteomic data evaluation. [file Data_Sheet_1.PDF]

Supplementary Material 1

1 Proteomic analysis

1.1 Protein digest

Protein samples were adjusted to final concentration of 0.5 µg/µL using 50 mM NH_4HCO_3, diluted by the same volume of 10% (w/v) sodium deoxycholate (SDC) in 50 mM NH_4HCO_3 (final extraction concentration was 5%) and incubated on ice for 20 min with intermittent vortexing. Proteins were then reduced with 10 mM dithiothreitol at 37°C for 60 min, alkylated with 20 mM iodoacetamide at RT for 30 min in darkness, and the unreacted iodoacetamide was quenched with further 10 mM dithiothreitol at RT for 15 min. The samples were diluted with 50 mM NH_4HCO_3 to decrease the concentration of SDC to 0.5% and digested with sequencing grade trypsin (Promega, #V5117) overnight at 37°C. SDC was removed following the modified phase transfer protocol (Masuda et al., 2008). Briefly, ethyl-acetate was added and the digested product was acidified by trifluoroacetic acid (TFA) to a final concentration of ca 2% (v/v). The mixtures were vortexed vigorously for 1 min, centrifuged at 14,000 × g for 5 min and the upper organic layer was removed. The extraction was repeated with fresh portion of ethyl-acetate. The aqueous phases were desalted on Empore™ C18-SD (4 mm/1 mL) extraction cartridges (Sigma Aldrich, #66871-U) and dried in vacuum.

1.2 LC-MS/MS analysis

LC-MS/MS analysis was performed on the Ultimate 3000 RSLCnano System (Dionex) coupled on-line through Nanospray Flex ion source with a Q Exactive mass spectrometer (Thermo Scientific). Peptide mixtures were dissolved in 2% acetonitrile / 0.05% TFA and loaded onto a capillary trap column (C18 PepMap100, 3 µm, 100 Å, 0.075 × 20 mm; Dionex) by 5 µL/min of 2% acetonitrile / 0.05% TFA for 5 min. Then they were separated on the capillary column (C18 PepMap RSLC, 2 µm, 100 Å, 0.075 × 150 mm; Dionex) by step linear gradient of mobile phase B (80% acetonitrile / 0.1% formic acid) over mobile phase A (0.1% formic acid) from 4% to 34% B in 68 min and from 34% to 55% B in 21 min at flow rate of 300 nL/min. The column was kept at 40°C and the eluent was monitored at 215 nm. Spraying voltage was 1.70 kV and heated capillary temperature was 275°C. The mass spectrometer was operated in the positive ion mode performing survey MS (at 350–1650 m/z) and data-dependent MS/MS scans of 12 most intense precursors with dynamic exclusion window of 60 s and isolation window of 1.6 Da. MS scans were acquired with resolution of 70,000 from 3×10^6 accumulated charges, and maximum fill time was 100 ms. Normalized collision energy for HCD fragmentation was 27 units. MS/MS spectra were acquired with resolution of 17,500 from 10^5 accumulated charges, and maximum fill time was 100 ms.

1.3 Protein identification and label-free quantification

Raw data sets were processed by MaxQuant ver. 1.6.2.3 coupled with Andromeda search engine (Cox and Mann, 2008). The reference proteome set of *F. tularensis* subsp. *holarctica* strain FSC200 was downloaded from NCBI in July 2017 (1438 sequences). MaxQuant-implemented database was used for the identification of common contaminants. The identification and quantification parameters of MaxQuant were set as follows: mass tolerance for the first search at 20 ppm, for the second search from recalibrated spectra at 4.5 ppm (with individual mass error filtering enabled); minimal peptide

length of 7 amino acids; maximal mass of peptide 4600 Da; fixed modification: carbamidomethylation of cysteine; variable modifications: oxidation of methionine and acetylation of protein N-term, maximum number of variable modifications: 5 per peptide; digestion with trypsin/P with maximum of 2 missed cleavages. Mass tolerance for fragments in MS/MS was 20 ppm, taking the 12 most intensive peaks per 100 Da for search. Minimal Andromeda score for modified peptides was 40 and minimal delta score for modified peptides was 6. False discovery rate (FDR) was based on the target decoy approach using reverted database and it was set to 0.01 for peptide and protein identification. For the identification of proteins in OMV samples, label-free quantification was disabled. For calculation of the protein abundances and relative comparison between OMV and membrane-enriched fraction as well as among the group of stress-derived OMV samples, the algorithm of relative intensity-based absolute quantification (riBAQ) was applied (Krey et al., 2014), matching between runs was enabled, and replicates were analyzed as separate experiments.

The mass spectrometry proteomics data have been deposited to the ProteomeXchange Consortium via the PRIDE (Perez-Riverol et al., 2019) partner repository with the dataset identifier PXD013074.

1.4 Proteomic data analysis

Downstream analysis was performed in Perseus software ver. 1.6.1.1 (Tyanova et al., 2016) on the data imported from the MaxQuant "proteinGroups.txt" output tables. The riBAQ algorithm was employed for the relative comparison between OMV and membranes and within the group of stress-derived OMV samples. The following workflow was used for comparison between OMV and membranes: The iBAQ values were imported, filtered from potential contaminants, reverse hits and proteins only identified by site, normalized to the sum of non-contaminant iBAQ per sample, and log2 transformed. The replicates were grouped, proteins with less than 2 peptides or with more than 1 missing valid value per group were filtered off and missing values were imputed (width 0.3, down shift 1.8, separately for each column). Relative protein quantity differences and statistical significances were calculated by two-sample Student's *t*-test with the truncation resulting from permutation-based FDR (FDR = 0.05, s0 = 2). Protein identification in OMV samples was performed by the same software. Proteins were filtered from potential contaminants; reverse hits and proteins only identified by site. A minimum of 2 peptides per protein in at least 3 replicates were required to accept a protein as successfully identified.

The comparison between different cultivation conditions was made according to a similar workflow, albeit with several differences: no missing value was allowed, so imputation was skipped; multiple sample test ANOVA (permutation-based FDR = 0.05, s0 = 2) was used to find proteins with significant changes between cultivation conditions. ANOVA-significant proteins were processed by the post-hoc test (FDR = 0.05). Hierarchical clustering was then applied on the post-hoc treated data of all ANOVA-significant proteins and ANOVA-significant OMV-enriched proteins. Enrichment analysis was performed using Fisher's exact test while using the Benjamini-Hochberg FDR = 0.02 for truncation.

1.5 Protein annotation

The proteins were sorted into functional categories based on Clusters of Orthologous Groups (COG) (Tatusov et al., 2000) and KEGG pathways as identified by BlastKOALA (Kanehisa et al., 2016) and by eggNOGG-mapper v.4.5 (Huerta-Cepas et al., 2016). Gene ontology terms and Hidden Markov

Models (HMM) were generated by eggNOGG-mapper v.4.5. Cellular localization of proteins was predicted by PSORTb v.3.0.2 (Yu et al., 2010).

Supplementary references

Cox, J., and Mann, M. (2008). MaxQuant enables high peptide identification rates, individualized p.p.b.-range mass accuracies and proteome-wide protein quantification. *Nat. Biotechnol.* 26, 1367–1372. doi:10.1038/nbt.1511.

Huerta-Cepas, J., Szklarczyk, D., Forslund, K., Cook, H., Heller, D., Walter, M. C., et al. (2016). eggNOG 4.5: a hierarchical orthology framework with improved functional annotations for eukaryotic, prokaryotic and viral sequences. *Nucleic Acids Res.* 44, D286–D293. doi:10.1093/nar/gkv1248.

Kanehisa, M., Sato, Y., and Morishima, K. (2016). BlastKOALA and GhostKOALA: KEGG tools for functional characterization of genome and metagenome sequences. *J. Mol. Biol.* 428, 726–731. doi:10.1016/j.jmb.2015.11.006.

Krey, J. F., Wilmarth, P. A., Shin, J.-B., Klimek, J., Sherman, N. E., Jeffery, E. D., et al. (2014). Accurate label-free protein quantitation with high- and low-resolution mass spectrometers. *J. Proteome Res.* 13, 1034–1044. doi:10.1021/pr401017h.

Masuda, T., Tomita, M., and Ishihama, Y. (2008). Phase transfer surfactant-aided trypsin digestion for membrane proteome analysis. *J. Proteome Res.* 7, 731–740. doi:10.1021/pr700658q.

Perez-Riverol, Y., Csordas, A., Bai, J., Bernal-Llinares, M., Hewapathirana, S., Kundu, D. J., et al. (2019). The PRIDE database and related tools and resources in 2019: improving support for quantification data. *Nucleic Acids Res.* 47, D442–D450. doi:10.1093/nar/gky1106.

Tatusov, R. L., Galperin, M. Y., Natale, D. A., and Koonin, E. V. (2000). The COG database: a tool for genome-scale analysis of protein functions and evolution. *Nucleic Acids Res.* 28, 33–36.

Tyanova, S., Temu, T., Sinitcyn, P., Carlson, A., Hein, M. Y., Geiger, T., et al. (2016). The Perseus computational platform for comprehensive analysis of (prote)omics data. *Nat. Methods* 13, 731–740. doi:10.1038/nmeth.3901.

Yu, N. Y., Wagner, J. R., Laird, M. R., Melli, G., Rey, S., Lo, R., et al. (2010). PSORTb 3.0: improved protein subcellular localization prediction with refined localization subcategories and predictive capabilities for all prokaryotes. *Bioinformatics* 26, 1608–1615. doi:10.1093/bioinformatics/btq249.
