# Supplementary material for: Francisella tularensis subsp. holarctica Releases Differentially Loaded Outer Membrane Vesicles Under Various Stress Conditions
Source: Front Microbiol. 2019 Oct 10;10:2304. doi: 10.3389/fmicb.2019.02304 (PMC6795709; doi:10.3389/fmicb.2019.02304)
Supplement: MATERIAL S2 — Notes to the OMV storage. [file Data_Sheet_2.PDF]

Supplementary Material 2

1 Supplementary notes on OMV storage

1.1 Storage conditions influence the shape and overall condition of OMV

In addition to the problems that come with low OMV yields from liquid media cultivation, there exists also another issue of proper handling and storage of OMV samples, and especially those intended for the study of OMV morphology by TEM. Storage influences the shape and overall condition of the vesicles. After long-term storage at +4°C or after freezing to −20°C, the nanotubes tended to loosen from their compact form and finally they disintegrated to form energetically more favorable spheres. Storage at −80°C seemed to be gentler and caused less damage to the tubes integrity, probably due to faster and more efficient freezing. **Supplementary Figure 2.1** shows the morphology of OMV after different storage times and conditions. Repeated freezing and thawing of OMV samples should definitely be avoided, and the storage conditions should be considered especially in the case of further biological functions studies.

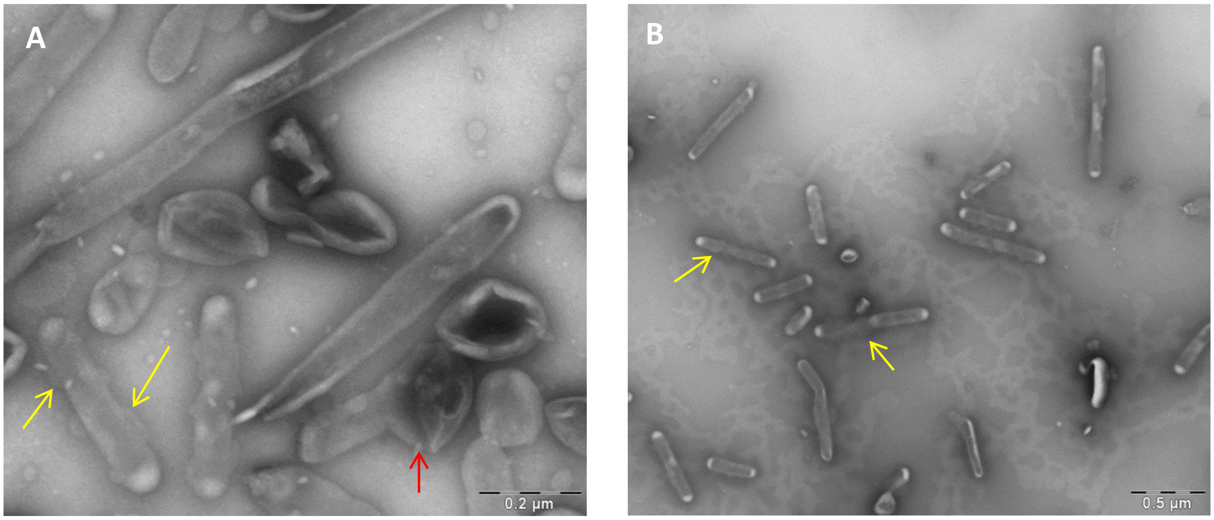

Supplementary Figure 2.1. Storage influences the shape and condition of the vesicles. (**A**) OMV sample was stored at −20°C for 14 days prior to TEM. The nanotubes tended to loosen from their compact form (yellow arrows point to the blebs that emerge on their surface) and finally the tubes “disintegrated” to form energetically more favorable spheres (red arrow). (**B**) OMV sample was stored at −80°C for 14 days prior to TEM.
