# Supplementary material for: Francisella tularensis subsp. holarctica Releases Differentially Loaded Outer Membrane Vesicles Under Various Stress Conditions
Source: Front Microbiol. 2019 Oct 10;10:2304. doi: 10.3389/fmicb.2019.02304 (PMC6795709; doi:10.3389/fmicb.2019.02304)
Supplement: MATERIAL S3 — Full list of all OMV-enriched proteins with references regarding their role in virulence. [file Data_Sheet_3.PDF]

Supplementary Material 3

1 Full list of proteins enriched in OMV in comparison with the membrane fraction. References regarding the proteins as virulence factor (VF), immunoreactive (IR), components of the capsule-like complex (CLC) or otherwise connected to virulence are cited.

Protein name	Gene name	FTS Locus tag	FTT Locus tag	FTL Locus tag	Enrichment [a]	Ref.
outer membrane protein of unknown function		FTS_0008	FTT_1747	FTL_0009	424.2	IR (Golovliov et al., 2013; Kilmury and Twine, 2011)
outer membrane protein OmpH	*ompH*	FTS_0538	FTT_1572c	FTL_0536	254.3	IR (Chandler et al., 2015; Kilmury and Twine, 2011)
hypothetical protein FTS_1462		FTS_1462	FTT_1334c	FTL_1494	246.4	CLC (Champion et al., 2018)
hypothetical protein FTS_1201		FTS_1201	FTT_0975	FTL_1225	126.9	IR (Chandler et al., 2015; Kilmury and Twine, 2011)
hypothetical protein FTS_1538		FTS_1538	FTT_0484	FTL_1579	126.5	secreted (Konecna et al., 2010), up-regulated in *dsbA* mutant (Straskova et al., 2009)
hypothetical protein FTS_0495		FTS_0495	FTT_0423	FTL_0493	102.0	up-regulated in *dsbA* mutant (Pavkova et al., 2017)
hypothetical protein FTS_0572		FTS_0572	FTT_1538c	FTL_0573	101.6	VF (Wallqvist et al., 2015)
hypothetical protein FTS_0814		FTS_0814	FTT_1137c	FTL_0822	73.0	
chitinase family 18 protein		FTS_1485	FTT_0715	FTL_1521	69.8	IR (Chandler et al., 2015), up-regulated *in vivo* in mice (Twine et al., 2006)
hypothetical protein FTS_0402		FTS_0402	FTT_1303c	FTL_0411	60.9	IR (Kilmury and Twine, 2011; Twine et al., 2010)
peroxidase/catalase	*katG*	FTS_1471	FTT_0721c	FTL_1504	56.7	VF (Binesse et al., 2015; Melillo et al., 2010), IR (Kilmury and Twine, 2011), secreted (Konecna et al., 2010)
histidine acid phosphatase		FTS_0029	---	FTL_0031	56.5	VF (Mohapatra et al., 2008)
beta-lactamase class A	*bla*	FTS_0870	FTT_0611c	FTL_0879	48.9	secreted (Konecna et al., 2010)
hypothetical protein FTS_1681		FTS_1681	FTT_0165c	FTL_1724	44.8	
hypothetical protein FTS_0755		FTS_0755	---	FTL_0755	38.4	
gamma-glutamyltranspeptidase	*ggt*	FTS_0764	FTT_1181c	FTL_0766	37.6	VF (Alkhuder et al., 2009)
rhodanese-like family protein		FTS_0824	FTT_1127	FTL_0834	35.5	
pyrrolidone carboxylylate peptidase	*pcp*	FTS_0203	FTT_0296	FTL_0207	35.5	IR (Havlasová et al., 2005)

hypothetical protein FTS_1495		FTS_1495	FTT_0704	FTL_1532	35.3	IR (Straskova et al., 2015), up-regulated in *dsbA* mutant (Pavkova et al., 2017)
hypothetical protein FTS_1272		FTS_1272	FTT_0364c	FTL_1299	34.1	
DNA topoisomerase I	*topA*	FTS_0417	FTT_0906c	FTL_0426	26.7	
protein-disulfide isomerase		FTS_1514	FTT_0507	FTL_1550	25.2	
exodeoxyribonuclease VII large subunit	*xseA*	FTS_0754	FTT_1190c	FTL_0754	24.5	
hypothetical protein FTS_0815		FTS_0815	FTT_1136c	FTL_0823	23.5	up-regulated in *dsbA* mutant (Pavkova et al., 2017)
hypothetical protein FTS_0974		FTS_0974	FTT_0540c	FTL_0994	23.4	up-regulated in *dsbA* mutant (Pavkova et al., 2017)
single-strand DNA binding protein	*ssb*	FTS_0013	FTT_1752	FTL_0014	23.4	IR (Havlasová et al., 2005)
outer membrane lipoprotein		FTS_1885	FTT_0211c	FTL_1939	21.9	
hypothetical protein FTS_0065		FTS_0065	FTT_1676	FTL_0073	20.4	VF (Rockx-Brouwer et al., 2012), IR (Janovská et al., 2007b)
hypothetical protein FTS_0452		FTS_0452	FTT_0385	FTL_0451	19.9	IR (Kilmury and Twine, 2011)
hypothetical protein FTS_0570		FTS_0570	FTT_1540c	FTL_0571	19.0	IR (Chandler et al., 2015; Kilmury and Twine, 2011)
hypothetical protein FTS_0659		FTS_0659	FTT_1402c	FTL_0661	18.9	up-regulated in *dsbA* mutant (Pavkova et al., 2017)
membrane protein of unknown function	*ttg2*	FTS_0515	FTT_1611	FTL_0514	18.3	
superoxide dismutase (Cu-Zn) precursor	*sodC*	FTS_0371	FTT_0879	FTL_0380	18.0	VF (Melillo et al., 2009)
hypothetical protein FTS_1749		FTS_1749	FTT_0066	FTL_1793	17.5	up-regulated in *dsbA* mutant (Pavkova et al., 2017)
FKBP-type peptidyl-prolyl cis-trans isomerase		FTS_1020	FTT_1043	FTL_1042	17.2	IR (Pavkova et al., 2006)
hypothetical protein FTS_0424		FTS_0424	FTT_0913	FTL_0434	16.4	
hypothetical protein FTS_0092		FTS_0092	FTT_1777c	FTL_0104	15.5	
hypothetical protein FTS_0571		FTS_0571	FTT_1539c	FTL_0572	15.5	IR (Kilmury and Twine, 2011), CLC (Champion et al., 2018)
hypothetical protein FTS_0317		FTS_0317	FTT_0825c	FTL_0317	15.0	
hypothetical protein FTS_0414		FTS_0414	FTT_0903	FTL_0423	14.6	IR (Straskova et al., 2015)
dGTP triphosphohydrolase	*dgt*	FTS_1470	FTT_0720c	FTL_1503	13.9	
peptide methionine sulfoxide reductase	*msrA*	FTS_1906	FTT_1797c	FTL_1960	13.4	up-regulated in macrophages (Wehrly et al., 2009)

hypothetical protein FTS_0493		FTS_0493	---	FTL_0491	13.1	
outer membrane lipoprotein	*blc*	FTS_1846	FTT_0198	FTL_1897	12.7	
group A colicin translocation tolB protein	*tolB*	FTS_0332	FTT_0840	FTL_0334	12.6	
OmpA family peptidoglycan-associated lipoprotein	*pal*	FTS_0334	FTT_0842	FTL_0336	12.5	secreted (Kinkead et al., 2018)
hypothetical protein FTS_1430		FTS_1430	FTT_0768c	FTL_1459	11.9	
VacJ like lipoprotein		FTS_1598	FTT_1591	FTL_1637	11.9	IR (Janovská et al., 2007a)
hypothetical protein FTS_1664		FTS_1664	FTT_1639c	FTL_1709	11.6	VF (Ren et al., 2014)
outer membrane lipoprotein	*omlA*	FTS_0061	FTT_1680c	FTL_0069	11.5	
hypothetical protein FTS_1187		FTS_1187	FTT_0989	FTL_1213	10.7	up-regulated in *dsbA* mutant (Pavkova et al., 2017), up-regulated in macrophages (Bent et al., 2013; Wehrly et al., 2009)
D-alanyl-D-alanine carboxypeptidase	*dacD*	FTS_1034	FTT_1029	FTL_1060	10.7	VF (Ren et al., 2014; Spidlova et al., 2018)
OmpA family protein	*fopA*	FTS_1295	FTT_0583	FTL_1328	10.6	VF (Rowe and Huntley, 2015), IR (Hickey et al., 2011)
Type IV pili, pilus assembly protein	*pilF*	FTS_1005	FTT_1057c	FTL_1029	10.6	VF (Ren et al., 2014)
OmpA family protein		FTS_0323	FTT_0831c	FTL_0325	10.5	VF (Robertson et al., 2014), IR (Kilmury and Twine, 2011)
hypothetical protein FTS_1068		FTS_1068	---	FTL_1097	9.9	down-regulated in *dsbA* mutant (Pavkova et al., 2017)
ATP-dependent Clp protease proteolytic subunit	*clpP*	FTS_0883	FTT_0624	FTL_0892	9.9	
hypothetical protein FTS_0983		FTS_0983	FTT_0550	FTL_1005	9.7	
outer membrane lipoprotein LolB	*lolB*	FTS_0143	FTT_0270	FTL_0150	9.6	
rare lipoprotein B family protein		FTS_1185	FTT_0991	FTL_1211	9.5	IR (Kilmury and Twine, 2011)
hypothetical protein FTS_0415		FTS_0415	FTT_0904	FTL_0424	9.0	
hypothetical protein FTS_1319		FTS_1319	FTT_0760c	---	9.0	
hypothetical protein FTS_1023		FTS_1023	FTT_1040	FTL_1045	8.7	
hypothetical protein FTS_0093		FTS_0093	FTT_1778c	FTL_0105	8.7	IR (Fulton et al., 2011; Golovliov et al., 2013; Post et al., 2017; Twine et al., 2010)
methionine sulfoxide reductase B	*msrB*	FTS_0370	FTT_0878c	FTL_0379	8.5	VF (Saha et al., 2017), up-regulated in macrophages (Wehrly et al., 2009)

hypothetical protein FTS_1229		FTS_1229	---	---	8.2	up-regulated in *dsbA* mutant (Pavkova et al., 2017)
hypothetical protein FTS_0642		FTS_0642	FTT_1416c	FTL_0645	8.0	IR (Post et al., 2017)
hypothetical protein FTS_0195		FTS_0195	FTT_0289c	FTL_0199	7.8	IR (Whelan et al., 2018)
hypothetical protein FTS_1076		FTS_1076	FTT_1095c	FTL_1107	7.2	
uroporphyrinogen decarboxylase	*hemE*	FTS_1769	FTT_0047	FTL_1812	6.7	
beta-lactamase class A	*blaA*	FTS_0937	FTT_0681c	FTL_0957	6.3	
membrane-bound lytic murein transglycosylase	*mltA*	FTS_1165	FTT_1271	FTL_1189	6.1	IR (Kilmury and Twine, 2011)
competence lipoprotein ComL	*comL*	FTS_0701	FTT_1244c	FTL_0700	6.1	up-regulated in macrophages (Bent et al., 2013)
X-prolyl aminopeptidase 2		FTS_0868	FTT_0609	FTL_0877	6.1	VF (Kadzhaev et al., 2009), up-regulated in macrophages (Wehrly et al., 2009)
hypothetical protein FTS_1279		FTS_1279	FTT_0369c	FTL_1306	6.1	VF (Rockx-Brouwer et al., 2012), IR (Straskova et al., 2015), up-regulated in *dsbA* mutant (Pavkova et al., 2017), up-regulated in macrophages (Bent et al., 2013)
hypothetical protein FTS_1540		FTS_1540	FTT_0482c	FTL_1581	5.9	VF (Horzempa et al., 2008; Wallqvist et al., 2015)
Type IV pili secretin component	*pilQ*	FTS_0792	FTT_1156c	FTL_0800	5.8	
hypothetical protein FTS_0163		FTS_0163	FTT_0243	FTL_0168	5.7	VF (Rohmer et al., 2007)
organic solvent tolerance protein, OstA	*ostA1*	FTS_1557	FTT_0467	FTL_1597	5.5	VF (Lai et al., 2010)
3-oxoacyl-(acyl-carrier-protein) reductase	*fabG*	FTS_1110	FTT_1375	FTL_1139	4.9	IR (Straskova et al., 2015), down-regulated in macrophages (Bent et al., 2013)
lipoprotein of unknown function	*lpnA*	FTS_0412	FTT_0901	FTL_0421	4.8	IR and protective (Forestal et al., 2008; Savitt et al., 2009; Sjöstedt et al., 1992; Thakran et al., 2008)
2-octaprenyl-3-methyl-6-methoxy-1,4-benzoquinol hydroxylase	*ubiF*	FTS_0727	FTT_1217c	FTL_0727	4.8	
outer membrane protein of unknown function		FTS_0537	FTT_1573c	FTL_0535	4.6	
hypothetical protein FTS_0685		FTS_0685	FTT_1260	FTL_0684	4.5	IR (Straskova et al., 2015)
hypothetical protein FTS_1018		FTS_1018	FTT_1045c	FTL_1040	4.4	
hypothetical protein FTS_1324		FTS_1324	FTT_0755	FTL_1359	4.3	
protein-disulfide isomerase		FTS_1067	FTT_1103	FTL_1096	4.1	VF and IR (Qin et al., 2011; Straskova et al., 2009, 2015)

hypothetical protein FTS_0283		FTS_0283	FTT_1506	FTL_0287	4.1	
phospholipase D family protein		FTS_1531	FTT_0490c	FTL_1570	4.0	up-regulated in macrophages (Raghunathan et al., 2010)
hypothetical protein FTS_0573		FTS_0573	FTT_1537c	FTL_0574	3.9	
hypothetical protein FTS_1129, FTS_0101		FTS_1129<br>FTS_0101	FTT_1355<br>FTT_1710	FTL_1161<br>FTL_0115	3.9	secreted into macrophages (Bröms et al., 2012)
hypothetical protein FTS_1139, FTS_0111		FTS_1139<br>FTS_0111	FTT_1346<br>FTT_1701	FTL_1170<br>FTL_0124	3.7	VF (Bröms et al., 2016; Robertson et al., 2013)
outer membrane protein tolC precursor	*tolC*	FTS_1817	FTT_1724c	FTL_1865	3.7	VF (Doyle et al., 2014; Gil et al., 2006)
hypothetical protein FTS_1178		FTS_1178	FTT_0998	FTL_1202	3.7	
chorismate mutase		FTS_0041	FTT_1650	FTL_0043	3.7	
serine-type D-Ala-D-Ala carboxypeptidase	*dacB*	FTS_1024	FTT_1039	FTL_1046	3.4	dsbA substrate (Qin et al., 2016)
hypothetical protein FTS_0568		FTS_0568	FTT_1542	FTL_0569	3.3	
TPR repeat-containing protein		FTS_0201	FTT_0294 [b]	FTL_0205	3.1	
hypothetical protein FTS_1845		FTS_1845	FTT_0199	FTL_1896	3.1	
sugar transamine/perosamine synthetase	*wbtI*	FTS_0600	FTT_1455c	FTL_0601	3.0	
malate dehydrogenase	*mdh*	FTS_0967	FTT_0535c	FTL_0987	2.9	IR (Kilmury and Twine, 2011), CLC (Champion et al., 2018)
hypothetical protein FTS_0037		FTS_0037	FTT_1653	FTL_0039	2.9	IR (Straskova et al., 2015)
glyceraldehyde-3-phosphate dehydrogenase/erythrose-4-phosphate dehydrogenase	*gapA*	FTS_1117	FTT_1368c	FTL_1146	2.8	VF, up-regulated in *dsbA* mutant, secreted (Pavkova et al., 2017)
ferrochelatase	*hemH*	FTS_0813	FTT_1138	FTL_0821	2.8	
hypothetical protein FTS_1135, FTS_0107		FTS_1135<br>FTS_0107	FTT_1349<br>FTT_1704	FTL_1167<br>FTL_0121	2.7	VF (Bröms et al., 2011; Lindgren et al., 2013; Rigard et al., 2016)
lipoate-protein ligase B	*lipB*	FTS_1032	FTT_1031	FTL_1058	2.7	
competence protein		FTS_1866	---	FTL_1916	2.7	
phosphoheptose isomerase	*gmhA*	FTS_0060	FTT_1681c	FTL_0068	2.7	
soluble lytic murein transglycosylase	*slt*	FTS_0467	FTT_0400	FTL_0466	2.7	

FAD binding family protein	*mreA*	FTS_0702	FTT_1243c	FTL_0701	2.6	
polar amino acid uptake transporter		FTS_0183	---	FTL_0187	2.6	
LysR family transcriptional regulator	*lysR*	FTS_0038	FTT_1652c	FTL_0040	2.6	
rRNA methyltransferase	*spoU*	FTS_1062	FTT_1108	FTL_1090	2.5	
glycerophosphoryl diester phosphodiesterase	*ugpQ*	FTS_1476	FTT_0726c	FTL_1511	2.5	IR (Kilmury and Twine, 2011)
glutamine amidotransferase subunit PdxT		FTS_1509	FTT_0512	FTL_1545	2.5	
membrane protein of unknown function		FTS_0430	FTT_0919	FTL_0439 [c]	2.4	
hypothetical protein FTS_1352		FTS_1352	FTT_0732	FTL_1384	2.4	
signal recognition particle GTPase	*ffh*	FTS_1213	FTT_0964c	FTL_1239	2.4	
glutamate-1-semialdehyde aminotransferase	*hemL*	FTS_1255	FTT_0927	FTL_1283	2.3	
DNA mismatch repair protein	*mutL*	FTS_1536	FTT_0486	FTL_1576	2.2	
U61 family peptidase		FTS_1636	FTT_0101	FTL_1678	2.2	IR (Kilmury and Twine, 2011), dsbA substrate (Qin et al., 2016; Ren et al., 2014)
membrane protein of unknown function		FTS_0429	FTT_0918	FTL_0439	2.2	VF (Salomonsson et al., 2009; Twine et al., 2005)
hypothetical protein FTS_1607		FTS_1607	FTT_0128	FTL_1646	2.2	
cytosol aminopeptidase	*pepA*	FTS_1445	FTT_1318c	FTL_1479	2.2	
histidine acid phosphatase		FTS_0996	FTT_1064	FTL_1021	2.2	VF (Mohapatra et al., 2013)
hypothetical protein FTS_0394		FTS_0394	---	FTL_0403	2.2	
hypothetical protein FTS_1048		FTS_1048	FTT_1015	FTL_1075	2.1	
elongation factor Ts	*tsf*	FTS_0222	FTT_0314	FTL_0225	2.1	IR (Golovliov et al., 2013; Kilmury and Twine, 2011)
outer membrane efflux protein		FTS_0687	FTT_1258	FTL_0686	2.1	VF (Alqahtani et al., 2018)
F0F1 ATP synthase subunit delta	*atpH*	FTS_1754	FTT_0061	FTL_1798	2.1	IR (Chandler et al., 2015)
dephospho-CoA kinase	*coaE*	FTS_0307	FTT_1487	FTL_0307	2.0	
P-pantothenate cysteine ligase/P-pantothenoylcysteine decarboxylase	*dfp*	FTS_0801	FTT_1147c	FTL_0808	2.0	
glycine dehydrogenase subunit 1	*gcvP1*	FTS_0481	FTT_0409	FTL_0479	2.0	up-regulated in macrophages (Wehrly et al., 2009)

glycosyl transferase, group 1	*waaG*	FTS_1403	FTT_0792	FTL_1429	1.9	
major facilitator transporter		FTS_1492	FTT_0708	FTL_1528	1.9	IR (Kilmury and Twine, 2011), up-regulated in macrophages (Wehrly et al., 2009)
hypothetical protein FTS_1593		FTS_1593	FTT_1596	FTL_1633	1.9	
hypothetical protein FTS_1258		FTS_1258	FTT_0924	FTL_1286	1.8	VF (Brunton et al., 2015)
sigma54 modulation protein	*yhbH*	FTS_1155	FTT_1281c	FTL_1179	1.8	IR (Kilmury and Twine, 2011)
potassium-transporting ATPase subunit A	*kdpA*	FTS_1832	---	FTL_1883	1.8	
S49 family serine peptidase		FTS_0007	FTT_1746	FTL_0008	1.8	
shikimate 5-dehydrogenase	*aroE*	FTS_0169	FTT_0238	FTL_0173	1.7	
preprotein translocase subunit SecG	*secG*	FTS_1734	FTT_0081	FTL_1779	1.7	
F0F1 ATP synthase subunit beta	*atpD*	FTS_1751	FTT_0064	FTL_1795	1.7	IR (Kilmury and Twine, 2011; Twine et al., 2010), CLC (Champion et al., 2018)
hypothetical protein FTS_0055		FTS_0055	FTT_1686c	FTL_0060	OMV[d]	
chitinase	*chiB*	FTS_0083	FTT_1768c	FTL_0093	OMV	
branched-chain amino acid aminotransferase protein (class IV)	*ilvE*	FTS_0122	FTT_0251	FTL_0131	OMV	
hypothetical protein FTS_0170		FTS_0170	FTT_0237c	FTL_0174	OMV	
hypothetical protein FTS_0282		FTS_0282	FTT_1507	FTL_0286	OMV	
hypothetical protein FTS_0297		FTS_0297	FTT_1493c	FTL_0300	OMV	
hypothetical protein FTS_0318		FTS_0318	FTT_0826c	FTL_0318	OMV	
hypothetical protein FTS_0333		FTS_0333	FTT_0841	FTL_0335	OMV	
NAD(P)H-dependent glycerol-3-phosphate dehydrogenase	*gpsA*	FTS_0363	FTT_0871	FTL_0372	OMV	
hypothetical protein FTS_0401		FTS_0401	FTT_1302	FTL_0410	OMV	
hypothetical protein FTS_0639		FTS_0639	---	FTL_0642	OMV	
hypothetical protein FTS_0738		FTS_0738	FTT_1206	FTL_0738	OMV	
methyltransferase		FTS_0765	---	FTL_0767	OMV	
radical SAM superfamily protein		FTS_1004	FTT_1058c	FTL_1028	OMV	
hypothetical protein FTS_1221		FTS_1221	FTT_0956c	FTL_1247	OMV	IR (Kilmury and Twine, 2011)
hypothetical protein FTS_1270		FTS_1270	FTT_0362c	FTL_1297	OMV	
hypothetical protein FTS_1318		FTS_1318	FTT_0761c	FTL_1353	OMV	

2-dehydro-3-deoxyphosphooctonate aldolase	*kdsA*	FTS_1499	FTT_0701	FTL_1535	OMV	
hypothetical protein FTS_1731		FTS_1731	FTT_0083	FTL_1776	OMV	IR (Kilmury and Twine, 2011)
aspartyl/glutamyl-tRNA amidotransferase subunit A	*gatA*	FTS_1797	FTT_0020	FTL_1842	OMV	

[a] OMV/membrane enrichment coefficient; [b] *FTT_0294* corresponds to the two distinct genes *FTS_0200 + FTS_0201* in FSC200 and it was erroneously annotated as a pseudogene in SchuS4 in the KEGG database (Dieppedale et al., 2013); [c] FTL_0439 in LVS is a fusion protein of FupA/B, in FSC200 and in SchuS4 it corresponds to two separate proteins (FTS_0429 and FTS_0430; FTT_0918, FTT_0919) (Siebert et al., 2019); [d] exclusively found in OMV and not in membrane fraction

2 References for Supplementary material 3

Alkhuder, K., Meibom, K. L., Dubail, I., Dupuis, M., and Charbit, A. (2009). Glutathione Provides a Source of Cysteine Essential for Intracellular Multiplication of Francisella tularensis. *PLoS Pathog.* 5. doi:10.1371/journal.ppat.1000284.

Alqahtani, M., Ma, Z., Ketkar, H., Suresh, R. V., Malik, M., and Bakshi, C. S. (2018). Characterization of a Unique Outer Membrane Protein Required for Oxidative Stress Resistance and Virulence of Francisella tularensis. *J. Bacteriol.* 200. doi:10.1128/JB.00693-17.

Bent, Z. W., Brazel, D. M., Tran-Gyamfi, M. B., Hamblin, R. Y., VanderNoot, V. A., and Branda, S. S. (2013). Use of a Capture-Based Pathogen Transcript Enrichment Strategy for RNA-Seq Analysis of the Francisella Tularensis LVS Transcriptome during Infection of Murine Macrophages. *PLoS ONE* 8. doi:10.1371/journal.pone.0077834.

Binesse, J., Lindgren, H., Lindgren, L., Conlan, W., and Sjöstedt, A. (2015). Roles of Reactive Oxygen Species-Degrading Enzymes of Francisella tularensis SCHU S4. *Infect. Immun.* 83, 2255–2263. doi:10.1128/IAI.02488-14.

Bröms, J. E., Lavander, M., Meyer, L., and Sjöstedt, A. (2011). IglG and IglI of the Francisella Pathogenicity Island Are Important Virulence Determinants of Francisella tularensis LVS ▿. *Infect. Immun.* 79, 3683–3696. doi:10.1128/IAI.01344-10.

Bröms, J. E., Meyer, L., and Sjöstedt, A. (2016). A mutagenesis-based approach identifies amino acids in the N-terminal part of Francisella tularensis IglE that critically control Type VI system-mediated secretion. *Virulence* 8, 821–847. doi:10.1080/21505594.2016.1258507.

Bröms, J. E., Meyer, L., Sun, K., Lavander, M., and Sjöstedt, A. (2012). Unique substrates secreted by the Type VI secretion system of Francisella tularensis during intramacrophage infection. *PLoS ONE* 7, e50473. doi:10.1371/journal.pone.0050473.

Brunton, J., Steele, S., Miller, C., Lovullo, E., Taft-Benz, S., and Kawula, T. (2015). Identifying Francisella tularensis Genes Required for Growth in Host Cells. *Infect. Immun.* 83, 3015–3025. doi:10.1128/IAI.00004-15.

Champion, A. E., Bandara, A. B., Mohapatra, N., Fulton, K. M., Twine, S. M., and Inzana, T. J. (2018). Further Characterization of the Capsule-Like Complex (CLC) Produced by Francisella tularensis Subspecies tularensis: Protective Efficacy and Similarity to Outer Membrane Vesicles. *Front. Cell. Infect. Microbiol.* 8. doi:10.3389/fcimb.2018.00182.

Chandler, J. C., Sutherland, M. D., Harton, M. R., Molins, ClaudiaR., Anderson, R. V., Heaslip, D. G., et al. (2015). Francisella tularensis LVS surface and membrane proteins as targets of effective post-exposure immunization for tularemia. *J. Proteome Res.* 14, 664–675. doi:10.1021/pr500628k.

Dieppedale, J., Gesbert, G., Ramond, E., Chhuon, C., Dubail, I., Dupuis, M., et al. (2013). Possible Links Between Stress Defense and the Tricarboxylic Acid (TCA) Cycle in Francisella Pathogenesis. *Mol. Cell. Proteomics MCP* 12, 2278–2292. doi:10.1074/mcp.M112.024794.

Doyle, C. R., Pan, J.-A., Mena, P., Zong, W.-X., and Thanassi, D. G. (2014). TolC-Dependent Modulation of Host Cell Death by the Francisella tularensis Live Vaccine Strain. *Infect. Immun.* 82, 2068–2078. doi:10.1128/IAI.00044-14.

Forestal, C. A., Gil, H., Monfett, M., Noah, C. E., Platz, G. J., Thanassi, D. G., et al. (2008). A conserved and immunodominant lipoprotein of Francisella tularensis is proinflammatory but not essential for virulence. *Microb. Pathog.* 44, 512–523. doi:10.1016/j.micpath.2008.01.003.

Fulton, K. M., Zhao, X., Petit, M. D., Kilmury, S. L. N., Wolfraim, L. A., House, R. V., et al. (2011). Immunoproteomic analysis of the human antibody response to natural tularemia infection with Type A or Type B strains or LVS vaccination. *Int. J. Med. Microbiol. IJMM* 301, 591–601. doi:10.1016/j.ijmm.2011.07.002.

Gil, H., Platz, G. J., Forestal, C. A., Monfett, M., Bakshi, C. S., Sellati, T. J., et al. (2006). Deletion of TolC orthologs in Francisella tularensis identifies roles in multidrug resistance and virulence. *Proc. Natl. Acad. Sci. U. S. A.* 103, 12897–12902. doi:10.1073/pnas.0602582103.

Golovliov, I., Twine, S. M., Shen, H., Sjostedt, A., and Conlan, W. (2013). A ΔclpB Mutant of Francisella tularensis Subspecies holarctica Strain, FSC200, Is a More Effective Live Vaccine than F. tularensis LVS in a Mouse Respiratory Challenge Model of Tularemia. *PLoS ONE* 8. doi:10.1371/journal.pone.0078671.

Havlasová, J., Hernychová, L., Brychta, M., Hubálek, M., Lenco, J., Larsson, P., et al. (2005). Proteomic analysis of anti-Francisella tularensis LVS antibody response in murine model of tularemia. *Proteomics* 5, 2090–2103. doi:10.1002/pmic.200401123.

Hickey, A. J., Hazlett, K. R. O., Kirimanjeswara, G. S., and Metzger, D. W. (2011). Identification of Francisella tularensis Outer Membrane Protein A (FopA) as a Protective Antigen for Tularemia. *Vaccine* 29, 6941–6947. doi:10.1016/j.vaccine.2011.07.075.

Horzempa, J., Carlson, P. E., O'Dee, D. M., Shanks, R. M., and Nau, G. J. (2008). Global transcriptional response to mammalian temperature provides new insight into Francisella tularensis pathogenesis. *BMC Microbiol.* 8, 172. doi:10.1186/1471-2180-8-172.

Janovská, S., Pávková, I., Hubálek, M., Lenčo, J., Macela, A., and Stulík, J. (2007a). Identification of immunoreactive antigens in membrane proteins enriched fraction from Francisella tularensis LVS. *Immunol. Lett.* 108, 151–159. doi:10.1016/j.imlet.2006.12.004.

Janovská, S., Pávková, I., Reichelová, M., Hubáleka, M., Stulík, J., and Macela, A. (2007b). Proteomic analysis of antibody response in a case of laboratory-acquired infection with Francisella tularensis subsp. tularensis. *Folia Microbiol. (Praha)* 52, 194–198.

Kadzhaev, K., Zingmark, C., Golovliov, I., Bolanowski, M., Shen, H., Conlan, W., et al. (2009). Identification of genes contributing to the virulence of Francisella tularensis SCHU S4 in a mouse intradermal infection model. *PLoS ONE* 4. doi:10.1371/journal.pone.0005463.

Kilmury, S. L. N., and Twine, S. M. (2011). The Francisella tularensis proteome and its recognition by antibodies. *Front. Microbiol.* 1. doi:10.3389/fmicb.2010.00143.

Kinkead, L. C., Whitmore, L. C., McCracken, J. M., Fletcher, J. R., Ketelsen, B. B., Kaufman, J. W., et al. (2018). Bacterial lipoproteins and other factors released by Francisella tularensis modulate human neutrophil lifespan: Effects of a TLR1 SNP on apoptosis inhibition. *Cell. Microbiol.* 20. doi:10.1111/cmi.12795.

Konecna, K., Hernychova, L., Reichelova, M., Lenco, J., Klimentova, J., Stulik, J., et al. (2010). Comparative proteomic profiling of culture filtrate proteins of less and highly virulent Francisella tularensis strains. *Proteomics* 10, 4501–4511. doi:10.1002/pmic.201000248.

Lai, X.-H., Shirley, R. L., Crosa, L., Kanistanon, D., Tempel, R., Ernst, R. K., et al. (2010). Mutations of Francisella novicida that Alter the Mechanism of Its Phagocytosis by Murine Macrophages. *PLoS ONE* 5. doi:10.1371/journal.pone.0011857.

Lindgren, M., Eneslätt, K., Bröms, J. E., and Sjöstedt, A. (2013). Importance of PdpC, IglC, IglI, and IglG for Modulation of a Host Cell Death Pathway Induced by Francisella tularensis. *Infect. Immun.* 81, 2076–2084. doi:10.1128/IAI.00275-13.

Melillo, A. A., Bakshi, C. S., and Melendez, J. A. (2010). Francisella tularensis antioxidants harness reactive oxygen species to restrict macrophage signaling and cytokine production. *J. Biol. Chem.* 285, 27553. doi:10.1074/jbc.M110.144394.

Melillo, A. A., Mahawar, M., Sellati, T. J., Malik, M., Metzger, D. W., Melendez, J. A., et al. (2009). Identification of Francisella tularensis live vaccine strain CuZn superoxide dismutase as critical for resistance to extracellularly generated reactive oxygen species. *J. Bacteriol.* 191, 6447–6456. doi:10.1128/JB.00534-09.

Mohapatra, N. P., Soni, S., Rajaram, M. V. S., Strandberg, K. L., and Gunn, J. S. (2013). Type A Francisella tularensis acid phosphatases contribute to pathogenesis. *PLoS ONE* 8. doi:10.1371/journal.pone.0056834.

Mohapatra, N. P., Soni, S., Reilly, T. J., Liu, J., Klose, K. E., and Gunn, J. S. (2008). Combined Deletion of Four Francisella novicida Acid Phosphatases Attenuates Virulence and Macrophage Vacuolar Escape. *Infect. Immun.* 76, 3690–3699. doi:10.1128/IAI.00262-08.

Pavkova, I., Kopeckova, M., Klimentova, J., Schmidt, M., Sheshko, V., Sobol, M., et al. (2017). The Multiple Localized Glyceraldehyde-3-Phosphate Dehydrogenase Contributes to the Attenuation of the Francisella tularensis dsbA Deletion Mutant. *Front. Cell. Infect. Microbiol.* 7. doi:10.3389/fcimb.2017.00503.

Pavkova, I., Reichelova, M., Larsson, P., Hubalek, M., Vackova, J., Forsberg, A., et al. (2006). Comparative proteome analysis of fractions enriched for membrane-associated proteins from Francisella tularensis subsp. tularensis and F. tularensis subsp. holarctica strains. *J. Proteome Res.* 5, 3125–3134. doi:10.1021/pr0601887.

Post, D. M. B., Slütter, B., Schilling, B., Chande, A. T., Rasmussen, J. A., Jones, B. D., et al. (2017). Characterization of Inner and Outer Membrane Proteins from Francisella tularensis Strains LVS and Schu S4 and Identification of Potential Subunit Vaccine Candidates. *mBio* 8. doi:10.1128/mBio.01592-17.

Qin, A., Scott, D. W., Rabideau, M. M., Moore, E. A., and Mann, B. J. (2011). Requirement of the CXXC Motif of Novel Francisella Infectivity Potentiator Protein B FipB, and FipA in Virulence of F. tularensis subsp. tularensis. *PLoS ONE* 6. doi:10.1371/journal.pone.0024611.

Qin, A., Zhang, Y., Clark, M. E., Moore, E. A., Rabideau, M. M., Moreau, G. B., et al. (2016). Components of the Type Six secretion system are substrates of Francisella tularensis Schu S4 DsbA-like FipB protein. *Virulence* 0, 00–00. doi:10.1080/21505594.2016.1168550.

Raghunathan, A., Shin, S., and Daefler, S. (2010). Systems approach to investigating host-pathogen interactions in infections with the biothreat agent Francisella. Constraints-based model of Francisella tularensis. *BMC Syst. Biol.* 4, 118. doi:10.1186/1752-0509-4-118.

Ren, G., Champion, M. M., and Huntley, J. F. (2014). Identification of disulfide bond isomerase substrates reveals bacterial virulence factors. *Mol. Microbiol.* 94, 926–944. doi:10.1111/mmi.12808.

Rigard, M., Bröms, J. E., Mosnier, A., Hologne, M., Martin, A., Lindgren, L., et al. (2016). Francisella tularensis IglG Belongs to a Novel Family of PAAR-Like T6SS Proteins and Harbors a Unique N-terminal Extension Required for Virulence. *PLoS Pathog.* 12. doi:10.1371/journal.ppat.1005821.

Robertson, G. T., Case, E. D. R., Dobbs, N., Ingle, C., Balaban, M., Celli, J., et al. (2014). FTT0831c/FTL_0325 Contributes to Francisella tularensis Cell Division, Maintenance of Cell Shape, and Structural Integrity. *Infect. Immun.* 82, 2935–2948. doi:10.1128/IAI.00102-14.

Robertson, G. T., Child, R., Ingle, C., Celli, J., and Norgard, M. V. (2013). IglE Is an Outer Membrane-Associated Lipoprotein Essential for Intracellular Survival and Murine Virulence of Type A Francisella tularensis. *Infect. Immun.* 81, 4026–4040. doi:10.1128/IAI.00595-13.

Rockx-Brouwer, D., Chong, A., Wehrly, T. D., Child, R., Crane, D. D., Celli, J., et al. (2012). Low Dose Vaccination with Attenuated Francisella tularensis Strain SchuS4 Mutants Protects against Tularemia Independent of the Route of Vaccination. *PLoS ONE* 7. doi:10.1371/journal.pone.0037752.

Rohmer, L., Fong, C., Abmayr, S., Wasnick, M., Larson Freeman, T. J., Radey, M., et al. (2007). Comparison of Francisella tularensis genomes reveals evolutionary events associated with the emergence of human pathogenic strains. *Genome Biol.* 8, R102. doi:10.1186/gb-2007-8-6-r102.

Rowe, H. M., and Huntley, J. F. (2015). From the outside-in: The Francisella tularensis envelope and virulence. *Front. Cell. Infect. Microbiol.* 5. doi:10.3389/fcimb.2015.00094.

Saha, S. S., Hashino, M., Suzuki, J., Uda, A., Watanabe, K., Shimizu, T., et al. (2017). Contribution of methionine sulfoxide reductase B (MsrB) to Francisella tularensis infection in mice. *FEMS Microbiol. Lett.* 364. doi:10.1093/femsle/fnw260.

Salomonsson, E., Kuoppa, K., Forslund, A.-L., Zingmark, C., Golovliov, I., Sjöstedt, A., et al. (2009). Reintroduction of Two Deleted Virulence Loci Restores Full Virulence to the Live Vaccine Strain of Francisella tularensis. *Infect. Immun.* 77, 3424–3431. doi:10.1128/IAI.00196-09.

Savitt, A. G., Mena-Taboada, P., Monsalve, G., and Benach, J. L. (2009). Francisella tularensis Infection-Derived Monoclonal Antibodies Provide Detection, Protection, and Therapy. *Clin. Vaccine Immunol. CVI* 16, 414–422. doi:10.1128/CVI.00362-08.

Siebert, C., Lindgren, H., Ferré, S., Villers, C., Boisset, S., Perard, J., et al. (2019). Francisella tularensis: FupA mutation contributes to fluoroquinolone resistance by increasing vesicle secretion and biofilm formation. *Emerg. Microbes Infect.* 8, 808–822. doi:10.1080/22221751.2019.1615848.

Sjöstedt, A., Sandström, G., and Tärnvik, A. (1992). Humoral and cell-mediated immunity in mice to a 17-kilodalton lipoprotein of Francisella tularensis expressed by Salmonella typhimurium. *Infect. Immun.* 60, 2855–2862.

Spidlova, P., Stojkova, P., Dankova, V., Senitkova, I., Santic, M., Pinkas, D., et al. (2018). Francisella tularensis D-Ala D-Ala Carboxypeptidase DacD Is Involved in Intracellular Replication and It Is Necessary for Bacterial Cell Wall Integrity. *Front. Cell. Infect. Microbiol.* 8. doi:10.3389/fcimb.2018.00111.

Straskova, A., Pavkova, I., Link, M., Forslund, A.-L., Kuoppa, K., Noppa, L., et al. (2009). Proteome Analysis of an Attenuated Francisella tularensis dsbA Mutant: Identification of Potential DsbA Substrate Proteins. *J. Proteome Res.* 8, 5336–5346. doi:10.1021/pr900570b.

Straskova, A., Spidlova, P., Mou, S., Worsham, P., Putzova, D., Pavkova, I., et al. (2015). Francisella tularensis type B ΔdsbA mutant protects against type A strain and induces strong inflammatory cytokine and Th1-like antibody response in vivo. *Pathog. Dis.* 73. doi:10.1093/femspd/ftv058.

Thakran, S., Li, H., Lavine, C. L., Miller, M. A., Bina, J. E., Bina, X. R., et al. (2008). Identification of Francisella tularensis lipoproteins that stimulate the toll-like receptor (TLR) 2/TLR1 heterodimer. *J. Biol. Chem.* 283, 3751–3760. doi:10.1074/jbc.M706854200.

Twine, S., Byström, M., Chen, W., Forsman, M., Golovliov, I., Johansson, A., et al. (2005). A Mutant of Francisella tularensis Strain SCHU S4 Lacking the Ability To Express a 58-Kilodalton Protein Is Attenuated for Virulence and Is an Effective Live Vaccine. *Infect. Immun.* 73, 8345–8352. doi:10.1128/IAI.73.12.8345-8352.2005.

Twine, S. M., Mykytczuk, N. C. S., Petit, M. D., Shen, H., Sjöstedt, A., Wayne Conlan, J., et al. (2006). In vivo proteomic analysis of the intracellular bacterial pathogen, Francisella tularensis, isolated from mouse spleen. *Biochem. Biophys. Res. Commun.* 345, 1621–1633. doi:10.1016/j.bbrc.2006.05.070.

Twine, S. M., Petit, M. D., Fulton, K. M., House, R. V., and Conlan, J. W. (2010). Immunoproteomics analysis of the murine antibody response to vaccination with an improved Francisella tularensis live vaccine strain (LVS). *PLoS ONE* 5. doi:10.1371/journal.pone.0010000.

Wallqvist, A., Memišević, V., Zavaljevski, N., Pieper, R., Rajagopala, S. V., Kwon, K., et al. (2015). Using host-pathogen protein interactions to identify and characterize Francisella tularensis virulence factors. *BMC Genomics* 16, 1106. doi:10.1186/s12864-015-2351-1.

Wehrly, T. D., Chong, A., Virtaneva, K., Sturdevant, D. E., Child, R., Edwards, J. A., et al. (2009). Intracellular biology and virulence determinants of Francisella tularensis revealed by transcriptional profiling inside macrophages. *Cell. Microbiol.* 11, 1128–1150. doi:10.1111/j.1462-5822.2009.01316.x.

Whelan, A. O., Flick-Smith, H. C., Homan, J., Shen, Z. T., Carpenter, Z., Khoshkenar, P., et al. (2018). Protection induced by a Francisella tularensis subunit vaccine delivered by glucan particles. *PLoS ONE* 13. doi:10.1371/journal.pone.0200213.
