# Supplementary material for: Francisella tularensis subsp. holarctica Releases Differentially Loaded Outer Membrane Vesicles Under Various Stress Conditions
Source: Front Microbiol. 2019 Oct 10;10:2304. doi: 10.3389/fmicb.2019.02304 (PMC6795709; doi:10.3389/fmicb.2019.02304)
Supplement: MATERIAL S4 — pH of BHI medium before and after cultivation of F. tularensis subsp. holarctica strain FSC200. [file Data_Sheet_4.PDF]

Supplementary Material 4

1 pH of BHI medium before and after cultivation of *F. tularensis* subsp. *holarctica* strain FSC200

pH of the medium before cultivation [a]	pH after 16 / 40 h of cultivation [b]
7.4	~7.5
6.8	~7.5
6.3	~7.0
5.8	~7.0
5.3	~6.0 (16 h) / ~7.0 (40 h)
4.8	~5.0
4.3	~4.5

[a] pH of BHI medium was adjusted with HCl.

[b] pH of the culture media after cultivation was checked using pH indicator strips (range 2.0–9.0, Macherey-Nagel, #92118) with accuracy of 0.5.
