# Supplementary material for: Francisella tularensis subsp. holarctica Releases Differentially Loaded Outer Membrane Vesicles Under Various Stress Conditions
Source: Front Microbiol. 2019 Oct 10;10:2304. doi: 10.3389/fmicb.2019.02304 (PMC6795709; doi:10.3389/fmicb.2019.02304)
Supplement: MATERIAL S5 — Exploratory analysis of the proteomic data – comparison of OMV isolated from different cultivation conditions. [file Data_Sheet_5.PDF]

Supplementary Material 5

1 Exploratory analysis of the proteomic data – comparison of OMV isolated from different cultivation conditions

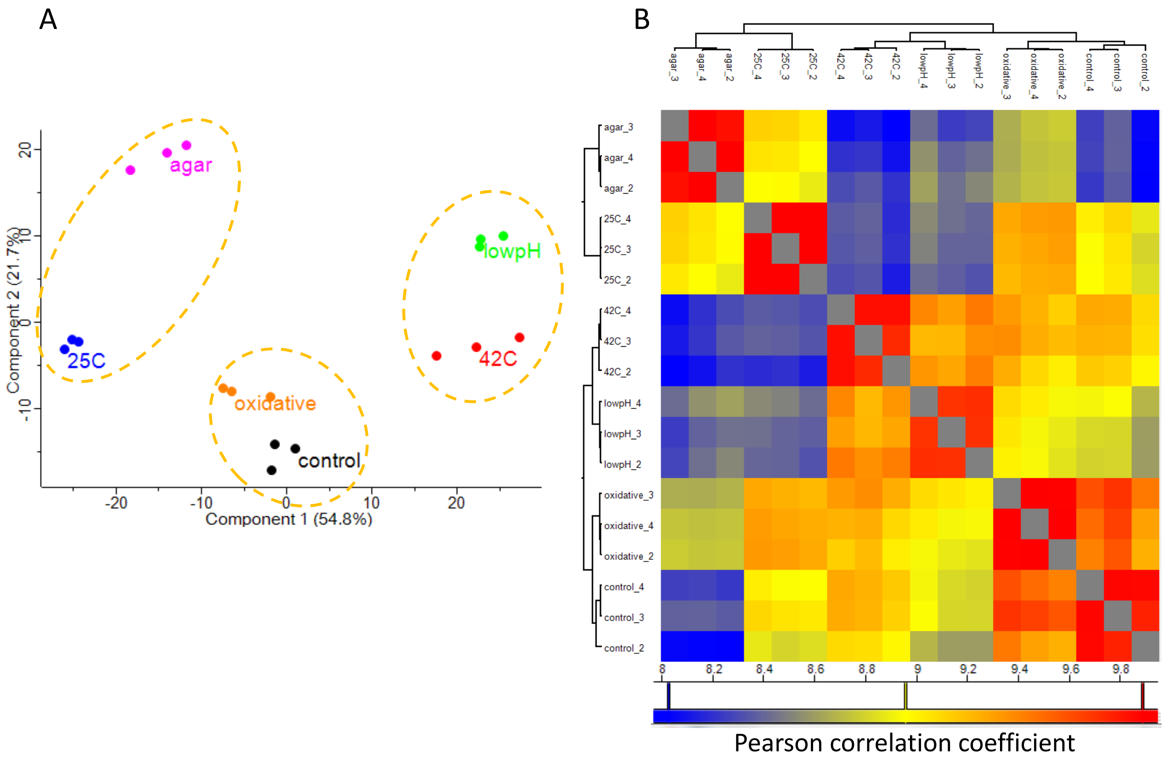

Supplementary Figure 5-1. Proteomic comparison of OMV from different cultivation conditions – exploratory analysis of the data. (**A**) Principal component analysis, and (**B**) hierarchical clustering of all the samples based on the Pearson correlation coefficients between them revealed highest similarity between control samples and those from oxidative stress. High temperature clustered well with low pH samples, and most different were the samples from low temperature and from agar plate.
