# Supplementary material for: Francisella tularensis subsp. holarctica Releases Differentially Loaded Outer Membrane Vesicles Under Various Stress Conditions
Source: Front Microbiol. 2019 Oct 10;10:2304. doi: 10.3389/fmicb.2019.02304 (PMC6795709; doi:10.3389/fmicb.2019.02304)
Supplement: MATERIAL S6 — Hierarchical clustering of the proteomic data – comparison of OMV isolated from different cultivation conditions. [file Data_Sheet_6.PDF]

frontiers

Supplementary Material 6

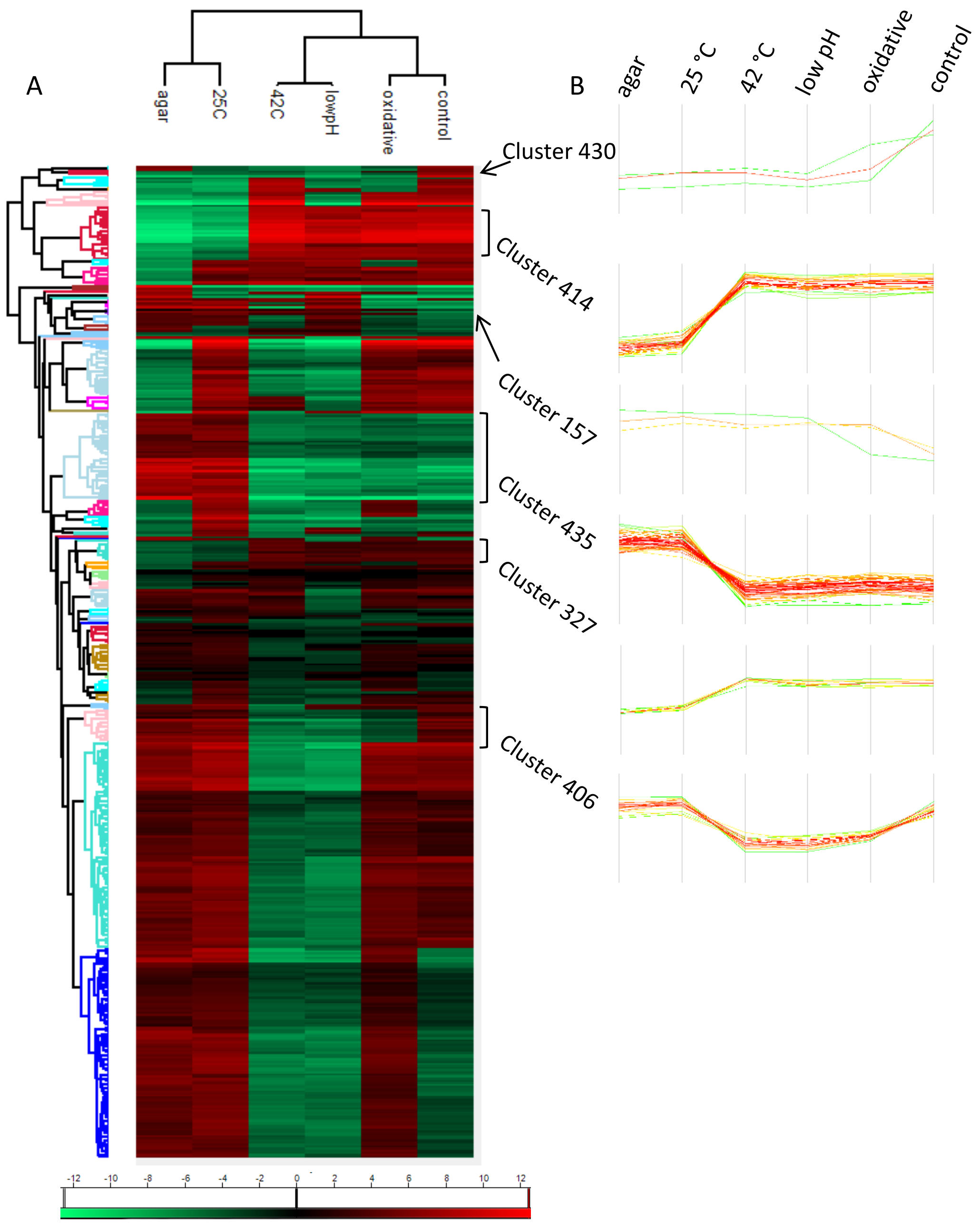

Supplementary Figure 6-1. (**A**) Hierarchical clustering of proteins with ANOVA-significantly different abundance in samples from different cultivation conditions. High and low abundances are shown in red and green, respectively. Clusters of proteins that are discussed in the text and listed in **Supplementary Table 6-1** are highlighted. (**B**) Profile plots of these clusters showing distinct behavior with respect to the cultivation conditions.

Supplementary Table 6-1. Protein clusters from the relative proteomic comparison of OMV isolated from six different cultivation conditions as depicted in **Supplementary Figure 6-1**. Hierarchical clustering was applied on all ANOVA-significant proteins. Enrichment factors and *p*-values of the enrichment were calculated by Fisher's exact test.

**Cluster 435**			
**enriched in:**	cytoplasmic localization (*p* = 8.7E-05, enrichment = 1.6)		
AFT93298.1	cell division protein	FTS_1660	ftsK
AFT92714.1	DNA/RNA endonuclease G	FTS_0869	
AFT93196.1	succinyl-CoA synthetase subunit beta	FTS_1517	sucC
AFT92111.1	chaperone clpB	FTS_0084	clpB
AFT92542.1	hypothetical protein FTS_0655	FTS_0655	
AFT92486.1	long chain fatty acid CoA ligase	FTS_0585	fadD
AFT93174.1	phosphopyruvate hydratase	FTS_1491	eno
AFT92917.1<br>AFT92129.1	hypothetical protein FTS_1134<br>hypothetical protein FTS_0106	FTS_1134<br>FTS_0106	
AFT92484.1	fusion product of 3-hydroxacyl-CoA dehydrogenase and acyl-CoA-binding protein	FTS_0583	fadB
AFT92731.1	host factor I for bacteriophage Q beta replication	FTS_0889	hfq
AFT92761.1	ribose-phosphate pyrophosphokinase	FTS_0930	prsA
AFT93367.1	glutaredoxin-related protein	FTS_1748	
AFT92806.1	AhpC/TSA family protein	FTS_0990	
AFT92911.1<br>AFT92123.1	intracellular growth locus protein D<br>intracellular growth locus protein D	FTS_1128<br>FTS_0100	iglD2<br>iglD1
AFT92905.1	pyruvate kinase	FTS_1119	pyk
AFT93128.1	purine nucleoside phosphorylase	FTS_1432	deoD
AFT93190.1	pyridoxal biosynthesis lyase PdxS	FTS_1510	
AFT93451.1	cell division protein FtsA	FTS_1858	ftsA
AFT93396.1	NADH dehydrogenase subunit G	FTS_1781	nuoG
AFT92397.1	BNR/Asp-box repeat-containing protein	FTS_0466	
AFT92509.1	thioredoxin	FTS_0610	trx1
AFT92815.1	50S ribosomal protein L9	FTS_1002	rplI
AFT92937.1	30S ribosomal protein S9	FTS_1162	rpsI
AFT92456.1	UDP-N-acetylglucosamine acyltransferase	FTS_0541	lpxA
AFT93359.1	succinate dehydrogenase iron-sulfur subunit	FTS_1740	sdhB
AFT92501.1	WbtJ protein	FTS_0601	wbtJ
AFT93305.1	chaperonin GroEL	FTS_1670	groEL
AFT93339.1	elongation factor Tu	FTS_1709	tufA
AFT93306.1	co-chaperonin GroES	FTS_1671	groES
AFT92942.1	molecular chaperone DnaK	FTS_1167	dnaK
AFT92466.1	signal peptidase I	FTS_0554	lepB
AFT93455.1	30S ribosomal protein S1	FTS_1862	rpsA
AFT92483.1	acetyl-CoA acetyltransferase	FTS_0582	fadA
AFT92960.1	hypothetical protein FTS_1195	FTS_1195	

AFT93007.1	hypothetical protein FTS_1258	FTS_1258	
AFT93450.1	cell division protein FtsZ	FTS_1857	ftsZ
AFT92159.1	universal stress protein	FTS_0161	uspA
AFT92895.1	acyl carrier protein	FTS_1109	acpP
AFT92222.1	30S ribosomal protein S10	FTS_0232	rpsJ
AFT92513.1	bacterioferritin	FTS_0617	bfr
AFT92077.1	HlyD family membrane fusion protein	FTS_0036	emrA2

Cluster 414

**enriched in:**	OMV-associated (*p* = 1.2E-19, enrichment = 5.1)<br>unknown localization (*p* = 5.8E-10, enrichment = 3.8)<br>periplasmic localization (*p* = 0.0022, enrichment = 6.2)		
AFT92267.1	hypothetical protein FTS_0282	FTS_0282	
AFT92666.1	hypothetical protein FTS_0814	FTS_0814	
AFT92476.1	hypothetical protein FTS_0571	FTS_0571	
AFT92632.1	gamma-glutamyltranspeptidase	FTS_0764	ggt
AFT93208.1	hypothetical protein FTS_1538	FTS_1538	
AFT92433.1	membrane protein of unknown function	FTS_0515	ttg2
AFT92161.1	hypothetical protein FTS_0163	FTS_0163	
AFT92965.1	hypothetical protein FTS_1201	FTS_1201	
AFT92326.1	methionine sulfoxide reductase B	FTS_0370	msrB
AFT92351.1	hypothetical protein FTS_0402	FTS_0402	
AFT93368.1	hypothetical protein FTS_1749	FTS_1749	
AFT92715.1	beta-lactamase class A	FTS_0870	bla
AFT92980.1	hypothetical protein FTS_1221	FTS_1221	
AFT93177.1	hypothetical protein FTS_1495	FTS_1495	
AFT92198.1	pyrrolidone carboxylylate peptidase	FTS_0203	pcp
AFT92477.1	hypothetical protein FTS_0572	FTS_0572	
AFT92421.1	hypothetical protein FTS_0495	FTS_0495	
AFT93352.1	hypothetical protein FTS_1731	FTS_1731	
AFT93439.1	hypothetical protein FTS_1845	FTS_1845	
AFT92071.1	histidine acid phosphatase	FTS_0029	
AFT93014.1	hypothetical protein FTS_1270	FTS_1270	
AFT93160.1	peroxidase/catalase	FTS_1471	katG
AFT92167.1	hypothetical protein FTS_0170	FTS_0170	
AFT93152.1	hypothetical protein FTS_1462	FTS_1462	
AFT92056.1	outer membrane protein of unknown function	FTS_0008	

Cluster 327

**enriched in:**	OMV-associated (*p* = 3.2E-07, enrichment = 5.1)		
AFT93482.1	peptide methionine sulfoxide reductase	FTS_1906	msrA
AFT93261.1	hypothetical protein FTS_1607	FTS_1607	
AFT92281.1	hypothetical protein FTS_0297	FTS_0297	
AFT92667.1	hypothetical protein FTS_0815	FTS_0815	
AFT92453.1	outer membrane protein OmpH	FTS_0538	ompH

AFT93168.1	chitinase family 18 protein	FTS_1485	
AFT93387.1	uroporphyrinogen decarboxylase	FTS_1769	hemE
AFT92110.1	chitinase	FTS_0083	chiB
AFT93224.1	organic solvent tolerance protein, OstA	FTS_1557	ostA1

Cluster 406

**enriched in:**	RNA polymerase (*p* = 6.98E-07, enrichment = 31.1)<br>Pyrimidine metabolism (*p* = 0.0001, enrichment = 12.5)<br>Purine metabolism (*p* = 0.0005, enrichment = 8.9)<br>GO:0006807 Nitrogen compound metabolism (*p* = 5.9E-05, enrichment = 3.4)		
AFT92923.1<br>AFT92135.1	PdpA protein<br>PdpA protein	FTS_1141<br>FTS_0113	pdpA2<br>pdpA1
AFT93442.1	glutamine synthetase	FTS_1848	glnA
AFT92331.1	aspartate aminotransferase	FTS_0376	aspC1
AFT93090.1	undecaprenyldiphospho-muramoylpentapeptide beta-N- acetylglucosaminyltransferase	FTS_1379	murG
AFT92219.1	30S ribosomal protein S12	FTS_0229	rpsL
AFT92919.1<br>AFT92131.1	hypothetical protein FTS_1137<br>hypothetical protein FTS_0109	FTS_1137<br>FTS_0109	
AFT93357.1	2-oxoglutarate dehydrogenase complex, E2 component, dihydrolipoyltranssuccinase	FTS_1738	sucB
AFT92851.1	1-deoxy-D-xylulose-5-phosphate synthase	FTS_1045	dxs
AFT93075.1	DEAD-box subfamily ATP-dependent helicase	FTS_1360	deaD
AFT93332.1	DNA-directed RNA polymerase subunit beta	FTS_1701	rpoB
AFT92660.1	P-pantothenate cysteine ligase/P-pantothenoylcysteine decarboxylase	FTS_0801	dfp
AFT93331.1	DNA-directed RNA polymerase, beta' subunit	FTS_1700	rpoC
AFT92248.1	DNA-directed RNA polymerase subunit alpha	FTS_0258	rpoA
AFT93333.1	50S ribosomal protein L7/L12	FTS_1702	rplL
AFT92511.1	DNA-directed RNA polymerase subunit alpha	FTS_0614	rpoA2

Cluster 157

AFT93475.1	putative ABC transporter ATP-binding protein	FTS_1893	
AFT92488.1	isocitrate dehydrogenase	FTS_0587	icd
AFT93218.1	acetyl-CoA carboxylase, biotin carboxylase subunit	FTS_1551	accC

Cluster 430

AFT92910.1<br>AFT92122.1	intracellular growth locus protein C<br>intracellular growth locus protein C	FTS_1127<br>FTS_0099	iglC2<br>iglC1
AFT92918.1<br>AFT92130.1	hypothetical protein FTS_1135<br>hypothetical protein FTS_0107	FTS_1135<br>FTS_0107	(iglG)
AFT93438.1<br>AFT93029.1<br>AFT92250.1	hypothetical protein FTS_1842<br>hypothetical protein FTS_1289<br>hypothetical protein FTS_0261	FTS_1842<br>FTS_1289<br>FTS_0261	

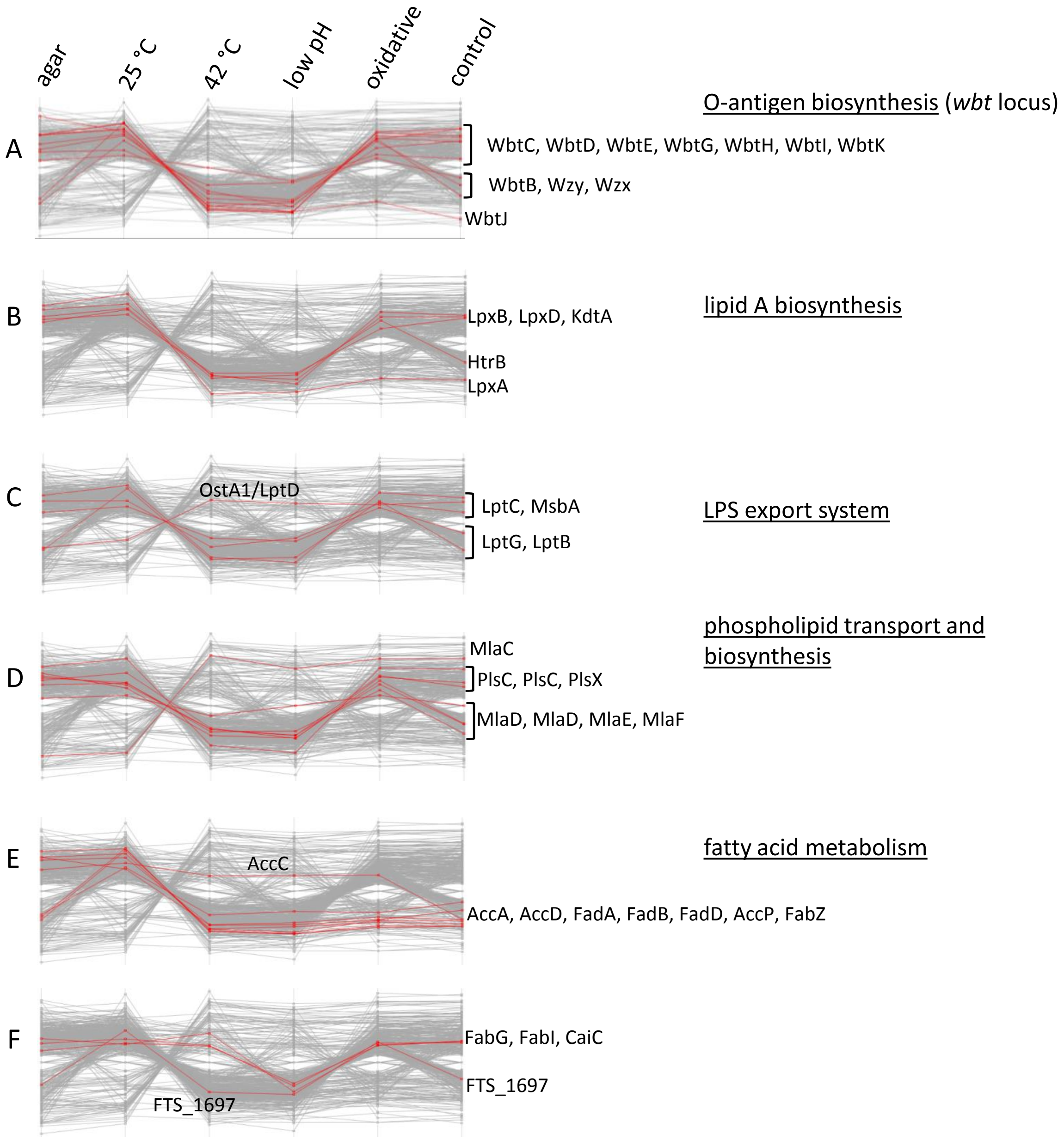

Supplementary Figure 6-2. Profile plots of selected biosynthetic pathways discussed in the text and listed in **Supplementary Table 6-2** on the background of all ANOVA-significant proteins.

Supplementary Table 6-2. List of proteins from biosynthetic pathways of LPS, phospholipids, and fatty acids depicted in **Supplementary Fig. 6-2**.

**O-antigen biosynthesis (*wbt* locus)**			
AFT92492.1	galactosyl transferase	FTS_0592	wbtB
AFT92493.1	UDP-glucose 4-epimerase	FTS_0593	wbtC
AFT92494.1	galacturonosyl transferase	FTS_0594	(wbtD)
AFT92495.1	UDP-glucose/GDP-mannose dehydrogenase	FTS_0595	wbtE
AFT92497.1	membrane protein/O-antigen protein	FTS_0597	wzy
AFT92498.1	hypothetical protein FTS_0598	FTS_0598	(wbtG)
AFT92499.1	glutamine amidotransferase/asparagine synthase	FTS_0599	wbtH
AFT92500.1	sugar transamine/perosamine synthetase	FTS_0600	wbtI
AFT92501.1	WbtJ protein	FTS_0601	wbtJ
AFT92502.1	O-antigen flippase	FTS_0602	(wzx)
AFT92503.1	glycosyltransferase	FTS_0603	wbtK
**Lipid A biosynthesis**			
AFT92456.1	UDP-N-acetylglucosamine acyltransferase	FTS_0541	lpxA
AFT92457.1	lipid-A-disaccharide synthase	FTS_0542	lpxB
AFT92454.1	UDP-3-O-[3-hydroxymyristoyl] glucosamine N-acyltransferase	FTS_0539	lpxD
AFT92462.1	3-deoxy-D-manno-octulosonic-acid transferase	FTS_0549	kdtA
AFT92173.1	LPS fatty acid acyltransferase	FTS_0176	htrB
**LPS export system**			
AFT92846.1	ABC transporter, ATP-binding protein	FTS_1039	yhbG (LptB)
AFT92844.1	hypothetical protein FTS_1037	FTS_1037	(lptC)
AFT93224.1	organic solvent tolerance protein, OstA	FTS_1557	ostA1 (lptD)
AFT93138.1	membrane protein of unknown function	FTS_1447	(lptG)
AFT93276.1	lipid exporter (LipidE) family protein	FTS_1627	msbA
**Phospholipid transport and biosynthesis**			
AFT92433.1	membrane protein of unknown function	FTS_0515	ttg2 (mlaC)
AFT92434.1	hypothetical protein FTS_0516	FTS_0516	(mlaD)
AFT92436.1	ABC transporter ATP-binding protein	FTS_0518	(mlaF)
AFT92576.1	hypothetical protein FTS_0696	FTS_0696	(mlaD)
AFT92578.1	ABC transporter membrane protein	FTS_0698	(mlaE)
AFT92106.1	acyltransferase	FTS_0079	(plsC)
AFT93458.1	putative acyltransferase	FTS_1865	plsC
AFT92899.1	putative glycerol-3-phosphate acyltransferase PlsX	FTS_1113	plsX
**Fatty acid metabolism**			

AFT92276.1	acetyl-CoA carboxylase carboxyltransferase subunit alpha	FTS_0292	accA
AFT93218.1	acetyl-CoA carboxylase, biotin carboxylase subunit	FTS_1551	accC
AFT93024.1	acetyl-CoA carboxylase, carboxytransferase subunit beta	FTS_1282	accD
AFT92483.1	acetyl-CoA acetyltransferase	FTS_0582	fadA
AFT92484.1	fusion product of 3-hydroxacyl-CoA dehydrogenase and acyl-CoA-binding protein	FTS_0583	fadB
AFT92486.1	long chain fatty acid CoA ligase	FTS_0585	fadD
AFT92895.1	acyl carrier protein	FTS_1109	acpP
AFT92894.1	beta-ketoacyl-ACP synthase II	FTS_1108	fabF
AFT92896.1	3-oxoacyl-(acyl-carrier-protein) reductase	FTS_1110	fabG
AFT93117.1	enoyl-ACP reductase I	FTS_1414	fabl
AFT92571.1	long chain fatty acid CoA ligase	FTS_0691	caiC
AFT93328.1	fatty acid desaturase	FTS_1697	

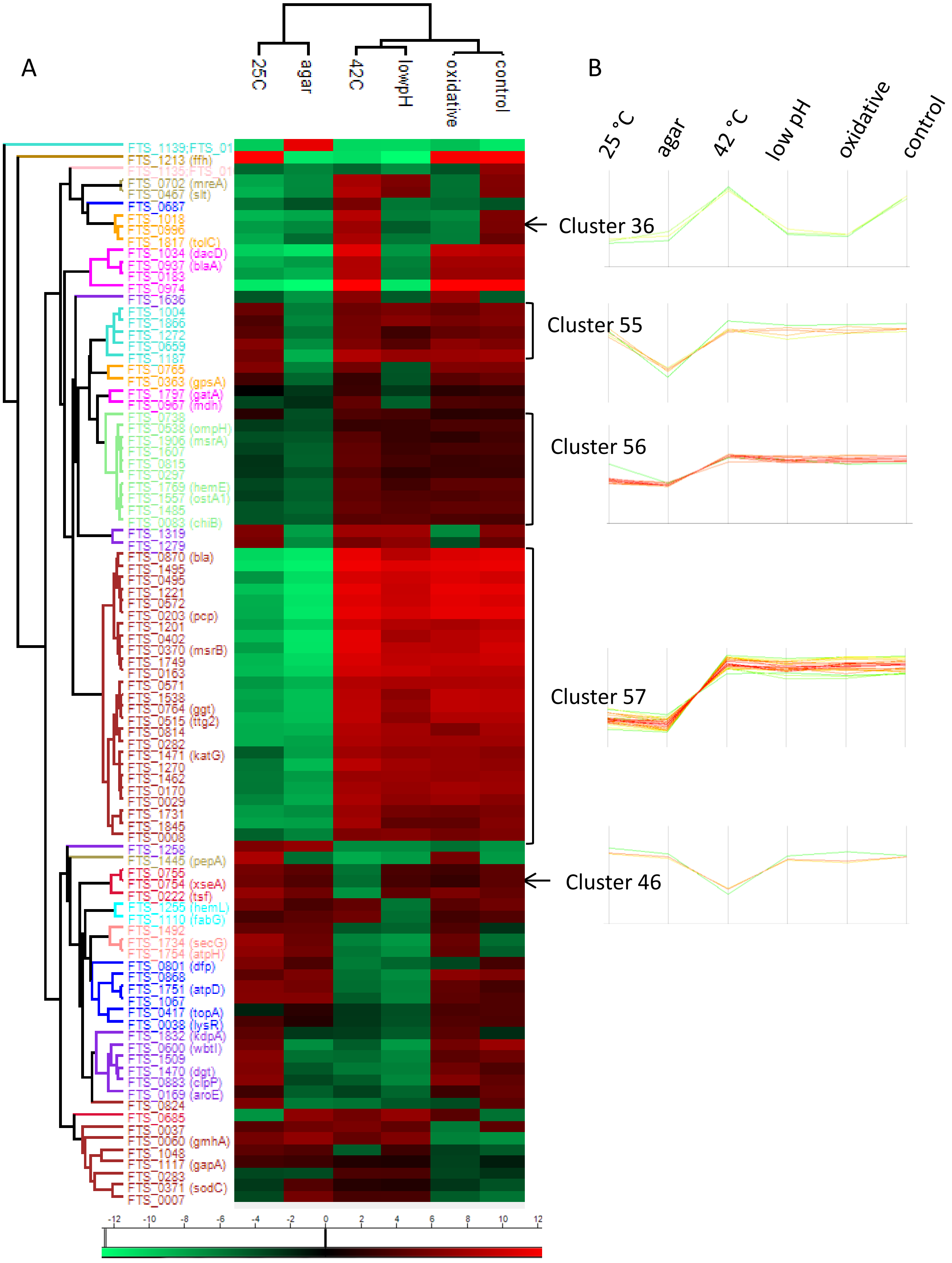

Supplementary Figure 6-3. (**A**) Hierarchical clustering of OMV-enriched, ANOVA-significant proteins. High and low abundances are shown in red and green, respectively. Clusters that are discussed in the text and listed in **Supplementary Table 6-3** are highlighted. (**B**) Profile plots of these clusters showing distinct behavior with respect to the cultivation conditions.

Supplementary Table 6-3. Protein clusters from the relative proteomic comparison of OMV isolated from six different cultivation conditions as depicted in **Supplemental Figure 6-3**. Hierarchical clustering was applied on ANOVA-significant proteins filtered on OMV-enriched proteins.

**Cluster 56**			
AFT92612.1	hypothetical protein FTS_0738	FTS_0738	
AFT92453.1	outer membrane protein OmpH	FTS_0538	ompH
AFT93482.1	peptide methionine sulfoxide reductase	FTS_1906	msrA
AFT93261.1	hypothetical protein FTS_1607	FTS_1607	
AFT92667.1	hypothetical protein FTS_0815	FTS_0815	
AFT92281.1	hypothetical protein FTS_0297	FTS_0297	
AFT93387.1	uroporphyrinogen decarboxylase	FTS_1769	hemE
AFT93224.1	organic solvent tolerance protein, OstA	FTS_1557	ostA1
AFT93168.1	chitinase family 18 protein	FTS_1485	(chiA)
AFT92110.1	chitinase	FTS_0083	chiB
**Cluster 57**			
AFT92715.1	beta-lactamase class A	FTS_0870	bla
AFT93177.1	hypothetical protein FTS_1495	FTS_1495	
AFT92421.1	hypothetical protein FTS_0495	FTS_0495	
AFT92980.1	hypothetical protein FTS_1221	FTS_1221	
AFT92477.1	hypothetical protein FTS_0572	FTS_0572	
AFT92198.1	pyrrolidone carboxylylate peptidase	FTS_0203	pcp
AFT92965.1	hypothetical protein FTS_1201	FTS_1201	
AFT92351.1	hypothetical protein FTS_0402	FTS_0402	
AFT92326.1	methionine sulfoxide reductase B	FTS_0370	msrB
AFT93368.1	hypothetical protein FTS_1749	FTS_1749	
AFT92161.1	hypothetical protein FTS_0163	FTS_0163	
AFT92476.1	hypothetical protein FTS_0571	FTS_0571	
AFT93208.1	hypothetical protein FTS_1538	FTS_1538	
AFT92632.1	gamma-glutamyltranspeptidase	FTS_0764	ggt
AFT92433.1	membrane protein of unknown function	FTS_0515	ttg2
AFT92666.1	hypothetical protein FTS_0814	FTS_0814	
AFT92267.1	hypothetical protein FTS_0282	FTS_0282	
AFT93160.1	peroxidase/catalase	FTS_1471	katG
AFT93014.1	hypothetical protein FTS_1270	FTS_1270	
AFT93152.1	hypothetical protein FTS_1462	FTS_1462	
AFT92167.1	hypothetical protein FTS_0170	FTS_0170	
AFT92071.1	histidine acid phosphatase	FTS_0029	
AFT93352.1	hypothetical protein FTS_1731	FTS_1731	
AFT93439.1	hypothetical protein FTS_1845	FTS_1845	
AFT92056.1	outer membrane protein of unknown function	FTS_0008	
**Cluster 55**			
AFT92817.1	radical SAM superfamily protein	FTS_1004	
AFT93459.1	competence protein	FTS_1866	

AFT93015.1	hypothetical protein FTS_1272	FTS_1272	
AFT92546.1	hypothetical protein FTS_0659	FTS_0659	
AFT92956.1	hypothetical protein FTS_1187	FTS_1187	

Cluster 46

AFT92624.1	hypothetical protein FTS_0755	FTS_0755	(xseB)
AFT92623.1	exodeoxyribonuclease VII large subunit	FTS_0754	xseA
AFT92212.1	elongation factor Ts	FTS_0222	tsf

Cluster 36

AFT92827.1	hypothetical protein FTS_1018	FTS_1018	
AFT92811.1	histidine acid phosphatase	FTS_0996	
AFT93426.1	outer membrane protein tolC precursor	FTS_1817	tolC
